# Functional roles of degraders and non-degraders in anaerobic trophic networks converting lignocellulose into monocarboxylates

**DOI:** 10.1038/s41522-026-01072-x

**Published:** 2026-06-24

**Authors:** Christina Schäfer, Maria L. Bonatelli, Idun Maria Tokvam Burgos, Sabine Kleinsteuber, Daniel Machado, Ove Øyås, Hauke Harms, Heike Sträuber

**Affiliations:** 1 Department of Microbial Biotechnology, Helmholtz Centre for Environmental Research – UFZ, Leipzig, Germany; 2 Department of Genetics, Martin Luther University Halle-Wittenberg, Halle, Germany; 3 Department of Biotechnology and Food Science Trondheim, Norwegian University of Science and Technology (NTNU), Trondheim, Norway; 4 Oslo Centre for Biostatistics and Epidemiology, Oslo University Hospital, Oslo, Norway; 5 Department of Applied Microbial Ecology, Helmholtz Centre for Environmental Research – UFZ, Leipzig, Germany

**Keywords:** Biotechnology, Microbiology

## Abstract

Lignocellulose is a promising renewable resource for anaerobic biochemical production, but its microbial conversion remains challenging. To elucidate metabolic networks in lignocellulose-degrading consortia, inocula of various origins were enriched on cellulose or xylan. Community composition and metabolic functions were revealed by amplicon sequencing, metagenomics, genome-scale metabolic modelling, and metabolic simulations. In cellulose-enriched communities, *Fibrobacter* and *Lacrimispora* consistently dominated as primary cellulose degraders, whereas *Bacteroides* likely functioned as secondary degraders. Acetic acid (up to 1.3 g l^-1^) and CO_2_ were the main fermentation products. Xylan enrichments produced C2-C6 fatty acids (up to 3.9 g l^-1^), lactic acid (up to 1.2 g l^-1^), ethanol (up to 1.2 g l^-1^), CO_2_, and H_2_. *Clostridium* dominated one xylan community and produced mainly butyric acid, while *Bifidobacterium* dominated another and produced mainly lactic acid. Caproic acid production was experimentally observed in one xylan enrichment. Metagenomic annotations and metabolic simulations suggest that *Lacrimispora amygdalina* degraded xylan and *Robinsoniella peoriensis* consumed xylobiose as a secondary consumer, both likely producing ethanol and lactic acid that supported caproic and butyric acid production by *Caproicibacter fermentans*. Integrated analysis identified functional guilds and clarified the roles of degraders and non-degraders, providing a blueprint for engineering synthetic consortia for sustainable biochemical production.

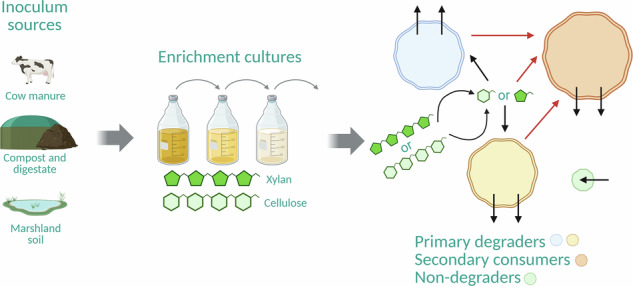

## Introduction

Humankind still relies heavily on fossil resources, which causes environmental impacts and drives the greatest challenges to humanity: climate change and loss of biodiversity. A circular economy based on renewable resources can reduce the pressure on climate and environment. Therefore, transformation of the economy must be fast-tracked, and organic waste must be utilised as a resource for renewable energy, materials, and chemicals. In order to manage the complexity of biowaste, it is necessary to integrate various types of conversion processes^1^.

Straw is such a complex biowaste, which is part of the largely unused quantities of agricultural waste worldwide^2^. The possibilities for treating large quantities of waste straw have so far been limited due to its complex chemical composition of 35–45% cellulose, 20–30% hemicellulose, and 8–15% lignin^3^. Cellulose is highly resistant to enzymatic hydrolysis due to its high crystallinity and interweaving with other polysaccharides^4^. Hemicellulose is easier to degrade, since it contains shorter-chain polysaccharides and the native form is amorphous. Hemicellulose can be divided into the four groups mannans, xyloglucans, mixed-linkage β-glucans, and xylans, the latter being the second most abundant plant biopolymer after cellulose^5^.

Prior to biotechnological utilisation of lignocellulose-rich waste, pretreatment methods such as organosolv, steam explosion, alkali treatment, ozonolysis, ammonium fibre explosion or acid treatments are often used. They all have in common that they are costly, rely on solvents or other chemicals, and produce inhibitors^6^. Biological pretreatment performed in a mild environment and without toxic by-products is environment-friendly and, if skilfully combined with the main conversion process, also cost-effective. One option of biological pretreatment is the use of purified enzymes such as cellulases (e.g., cellobiohydrolase, endoglucanases, β-glucosidase) and xylanases (e.g., endo-1,4-β-xylanase, 1,4-β-xylosidase)^7–9^. However, employing microorganisms instead of expensive enzymes, chemicals or mechanical equipment is technically more feasible and economically advantageous^10^.

Microbial communities surpass the capabilities of pure cultures by providing a rich repertoire of enzymes and pathways that complement each other in their function to efficiently use lignocellulose as a substrate, and can therefore deal with the complexity of lignocellulose^11,12^. Besides their extended hydrolytic functions, fermentative microbial communities can be used to produce chemicals that are currently still obtained from fossil raw materials or non-sustainably from palm oil. Hydrolysis and fermentation can take place in the same process, which has a favourable effect on economic efficiency. The products include alcohols (e.g., ethanol), energy-rich gases (methane or hydrogen), short-chain carboxylic acids (SCCAs; e.g., lactic, acetic or butyric acid) or medium-chain carboxylic acids (MCCAs; e.g., caproic or caprylic acid)^13^. MCCAs are produced by microbial chain elongation in which the reverse β-oxidation pathway is active. In this process, an electron donor, such as ethanol or lactate, is coupled with SCCAs (e.g., acetic acid) to produce longer-chain carboxylic acids, such as butyric, valeric, caproic, and caprylic acid^14,15^. Producing SCCAs and MCCAs from a renewable and abundant resource, such as lignocellulosic waste and residues, by microbial communities is probably the most environment-friendly way. Targeted chemical production requires knowledge-based process control, which is based on fundamental understanding of the metabolic networks.

The aim of this study was to enrich anaerobic microbial communities that can degrade cellulose or hemicellulose, as the main components of lignocellulosic biomass, producing SCCAs and MCCAs, and to reveal their metabolic network. Cow manure, marshland soil, and a mixture of compost and digestate from a biogas reactor were used as inoculum sources. The enriched consortia were batch-cultured in successive transfers with amorphous or crystalline cellulose, or with xylan, which was used as model substance for hemicellulose. The enrichment of specific bacterial taxa in the individual transfers was monitored by amplicon sequencing of 16S rRNA genes, while the functional potential of the enriched consortia was revealed by metagenome analyses. Metagenome-assembled genomes (MAGs) and the experimental data were used to build genome-scale metabolic models (GEMs). To obtain a deeper mechanistic insight into the role of community members and their interactions, community simulations were performed to elucidate cross-feeding interactions reflected in the experimentally observed production. We particularly focussed on understanding the roles of degraders and non-degraders in the assembly of stable enriched consortia, and the contributions of community members with very different abundance profiles.

## Results

### Effect of inoculum source and substrate on microbial community structure and metabolite production

Metabolic performance of the controls without the addition of cellulose/hemicellulose is shown in the Supplementary Figs. 1, 2. Only compounds >100 mg l^-1^ are displayed in the main figures, while information on lower concentrated compounds is included in the Supplementary Datasets 1-3.

The cellulose cultures produced CO_2_, with headspace proportions ranging from 1.1 to 21.7%, while the gas composition otherwise changed only marginally. In contrast, the xylan cultures additionally produced H_2_ at concentrations ranging from 1.3 to 47.5% in the headspace (Supplementary Figs. 3–5).

**Fig. 1:**
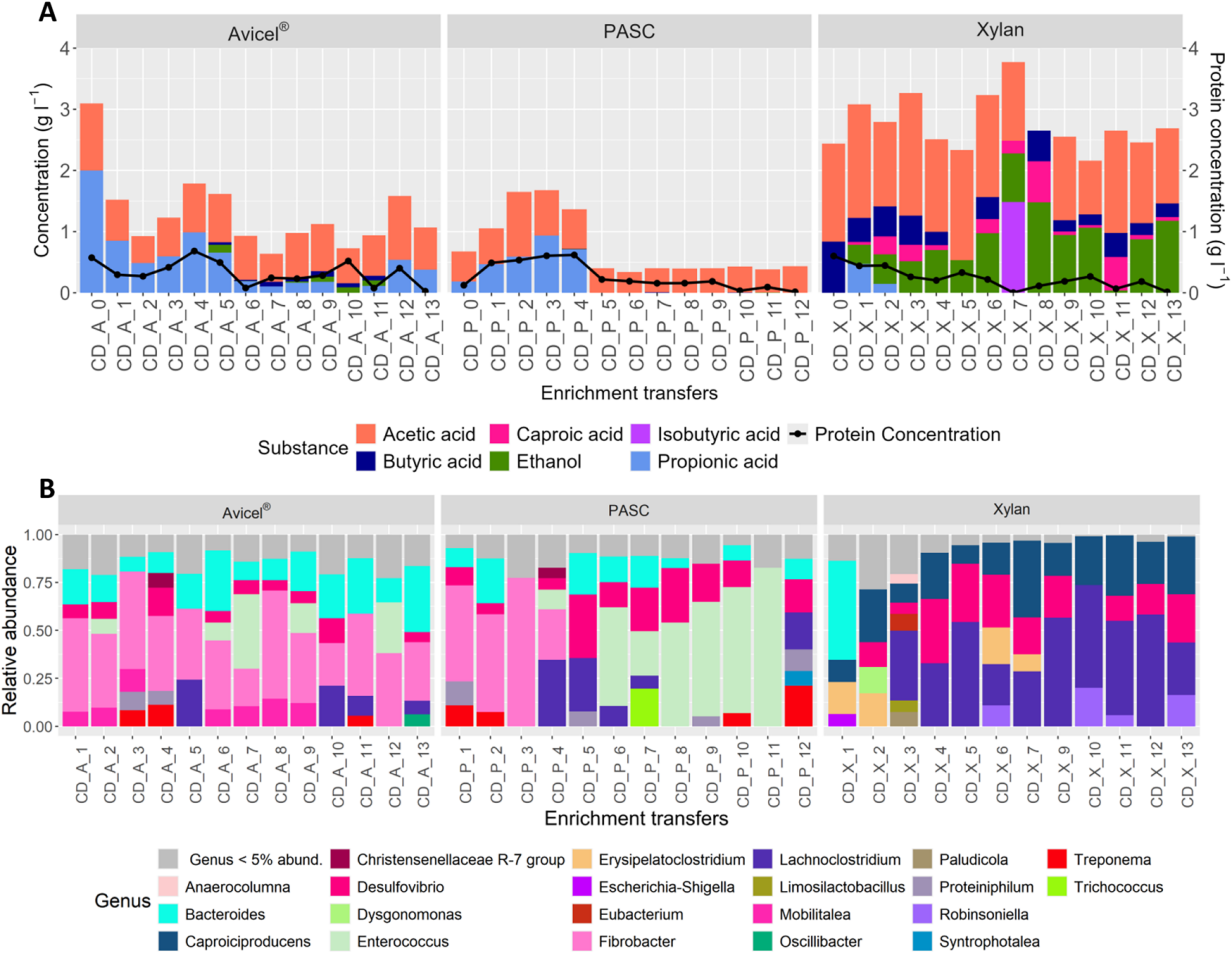
Enrichment cultures with compost and digestate as inoculum. **A** Metabolic products and protein concentrations, and **B** corresponding community compositions of the cultures grown on Avicel®, PASC, or xylan are shown. Every bar represents a distinct serial batch transfer (i.e. one enrichment cultivation cycle).

**Fig. 2:**
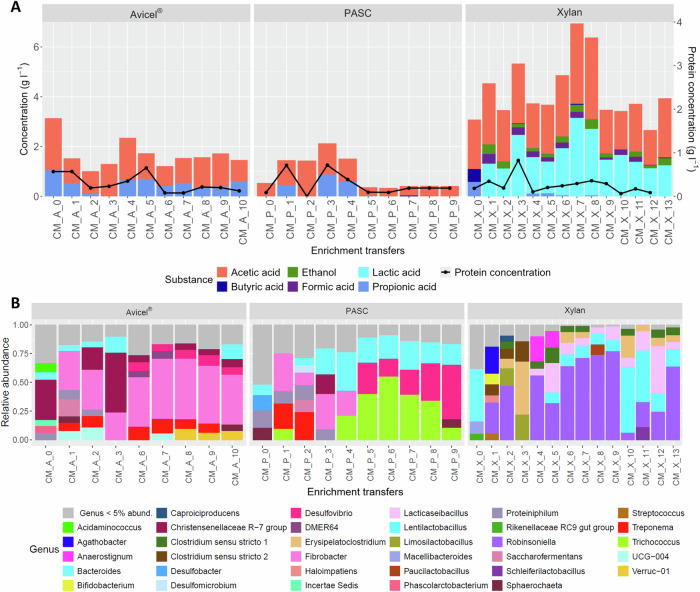
Enrichment cultures with cow manure as inoculum. **A** Metabolic products and protein concentrations, and **B** corresponding community compositions of the cultures grown on Avicel®, PASC, or xylan are shown. Every bar represents a distinct serial batch transfer (i.e. one enrichment cultivation cycle). SynCon2 medium was used for the cultures with Avicel® from transfer number 5 onwards. Verruc-01 belongs to the phylum Verrucomicrobiota. UCG-004 belongs to the class Clostridia. DMER64 belongs to the phylum Actinobacteriota.

**Fig. 3:**
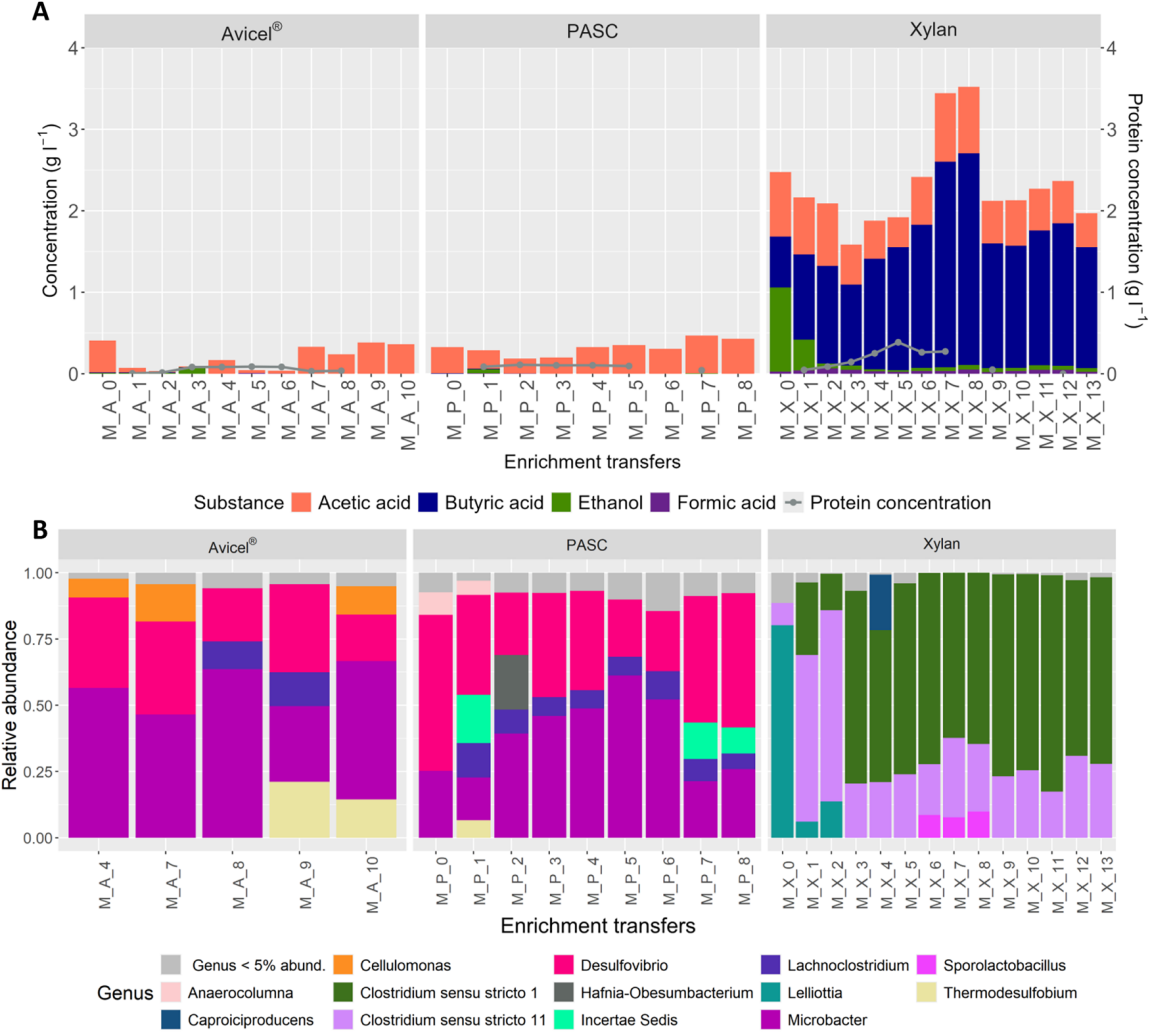
Enrichment cultures with marshland soil as inoculum. **A** Metabolic products and protein concentrations, and **B** corresponding community compositions of the cultures grown on Avicel®, PASC, or xylan are shown. Every bar represents a distinct serial batch transfer (i.e. one enrichment cultivation cycle). Missing values for community composition and protein concentration were due to the cultures having too low growth for DNA isolation and/or protein concentration measurement.

Cellulose enrichment cultures produced mainly acetic acid (Figs. 1A, 2A, 3A), and with compost and digestate or cow manure as inoculum additionally propionic acid. Product concentrations were consistently higher with Avicel® compared to PASC, except for marshland soil cultures, which yielded more products from PASC. Using SynCon2 medium enhanced product formation in the Avicel® enrichment from cow manure, in contrast to the other restarted enrichments (Supplementary Fig. 6). ASV data of the cultures enriched from compost and digestate or cow manure showed that *Fibrobacter* and *Bacteroides* predominated in the Avicel® cultures (Figs. 1B, 2B). Additionally, *Desulfovibrio* was dominant at the end of the transfers in all cultures with PASC as substrate (Figs. 1B, 2B, 3B).

When xylan was used as substrate, several other products such as butyric acid, isobutyric acid, and substantial amounts of caproic acid were detected in the compost and digestate enrichment cultures (Fig. 1A). Cow manure enrichments produced lactic acid, and marshland soil enrichments produced butyric acid in high concentrations throughout the whole process (Figs. 2A, 3A). From the 3^rd^ transfer, *Lachnoclostridium*, *Desulfovibrio*, and *Caproiciproducens* were consistently observed in the xylan cultures from compost and digestate (Fig. 1B). In the final cow manure transfers, *Robinsoniella*, *Lentilactobacillus*, *Lacticaseibacillus*, Puniceicoccaceae genus Verruc-01, and *Clostridium* sensu stricto 1 were the most abundant bacterial genera (Fig. 2B). The community composition of the cultures from marshland soil was fairly stable throughout the enrichment process, with *Clostridium* sensu stricto 1 and *Clostridium* sensu stricto 11 being most abundant (Fig. 3B).

After 2 weeks of cultivation, xylan was almost completely degraded in cultures from compost and digestate or marshland soil, while cultures from cow manure degraded about 70%. In contrast, after five weeks, Avicel® degradation remained < 20% in cultures inoculated with compost and digestate or cow manure (Supplementary Fig. 7).

Spearman correlations between metabolite concentrations and the relative abundances of the top 20 ASVs suggest that *Fibrobacter* (ASV1) was associated with propionic acid production, while *Clostridium* sensu stricto 1 abundance correlated positively with formic and butyric acid production. Lactic acid production correlated positively with *Robinsoniella* and *Erysipelatoclostridium* abundances, while caproic acid production correlated positively with the relative abundances of *Lachnoclostridium*, *Caproiciproducens*, and *Erysipelatoclostridium* (Supplementary Fig. 8).

### Phylogenomic diversity and metabolic potential

**Fig. 4:**
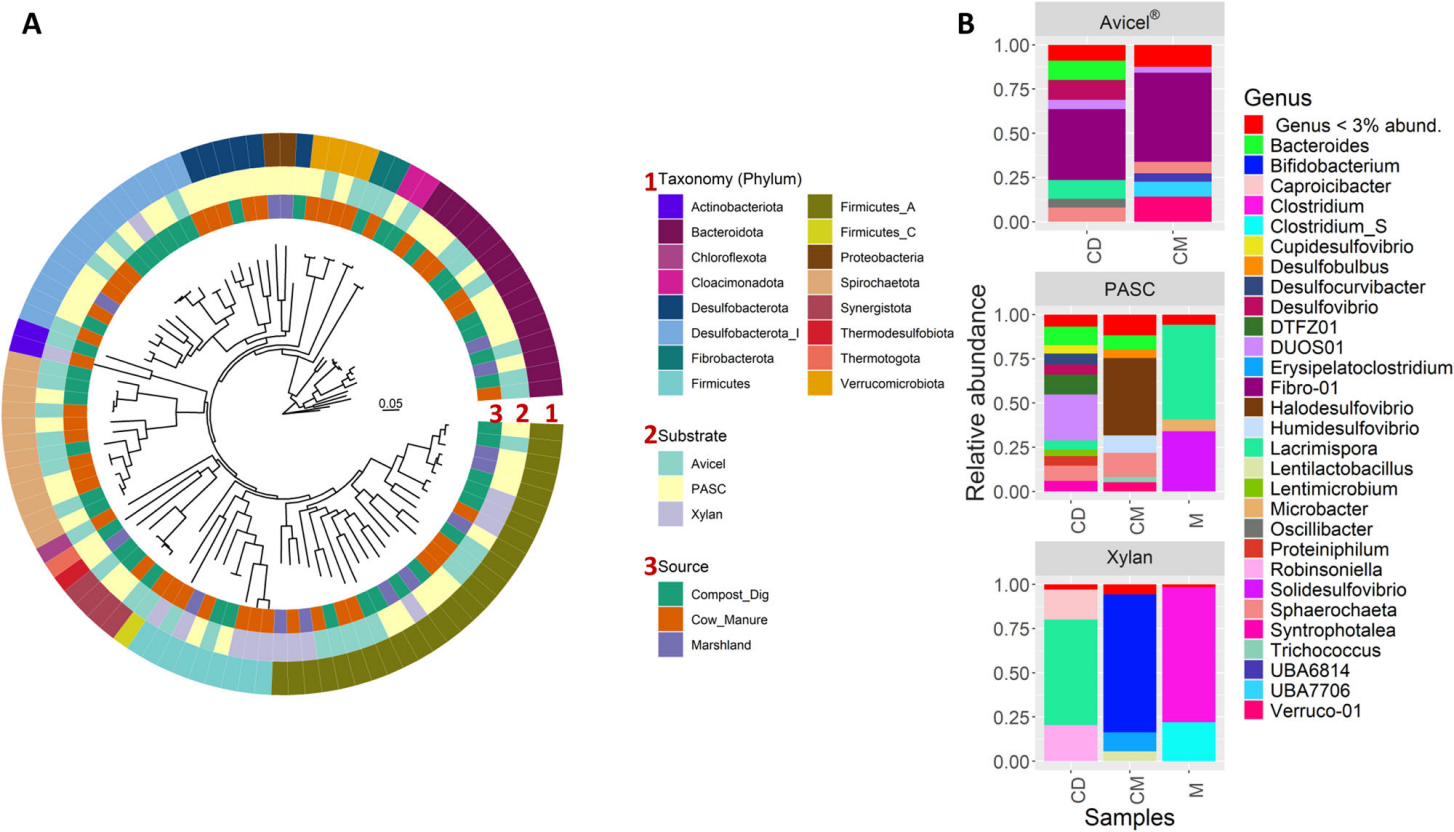
Taxonomic diversity of the metagenome-assembled genomes (MAGs) recovered from the enrichment cultures. **A** Phylogenomic tree of the MAGs reconstructed using the PhyloPhlAn database based on 400 universal marker genes^89^. **B** Community composition of the enriched consortia at the genus level. CD compost and digestate, CM cow manure, M marshland soil. DTFZ01 belongs to the order Christensenellales. DUOS01 belongs to the family Treponemataceae. Fibro-01 belongs to the family Fibrobacteraceae. UBA6814 belongs to the family Desulfovibrionaceae. UBA7706 belongs to the class Bacilli. Verruc-01 belongs to the phylum Verrucomicrobiota.

The enrichment process reduced the bacterial diversity found in the inocula (Supplementary Fig. 9A). Ordination of the ASV data showed a clear clustering, reflecting the origin of the inoculum and the substrate used, which was confirmed by PERMANOVA (Supplementary Fig. 9B). Metagenomic analysis yielded 102 high-quality MAGs across all enrichment cultures (Supplementary Dataset 4). The sum of the relative abundances of each MAG recovered from every enrichment culture ranged from 87.17% to 97.07% (Supplementary Dataset 5), confirming that the dominant taxa were well represented in the reconstructed genomes. Diversity patterns derived from MAGs mirrored those of the ASV data, both in terms of alpha diversity and sample ordination (Supplementary Figs. 9 and 10). At the phylum level, both methods revealed similar taxonomic profiles, with *Firmicutes*, *Desulfobacterota*, and *Bacteroidota* dominating the enriched communities (Fig. 4A, Supplementary Fig. 11), underscoring the robustness of the taxonomic assignments across methods.

The distribution of the different phyla in the enrichment cultures shows that *Firmicutes* was the only phylum present in all enrichment cultures, while *Desulfobacterota* and *Bacteroidota* were present mainly in cellulose enrichment cultures (Fig. 4A). Regarding the community composition at the genus level, enrichments with cellulose showed higher diversity compared to the xylan enrichments, with Fibro-01 (Fibrobacteraceae) being predominant in Avicel® cultures, and *Lacrimispora*, *Solidesulfovibrio*, *Halodesulfovibrio*, and DUOS01 (Treponemataceae) dominating in PASC cultures. The predominant genera in enrichment cultures grown on xylan were *Lacrimispora*, *Bifidobacterium*, and *Clostridium*, respectively (Fig. 4B).

**Fig. 5:**
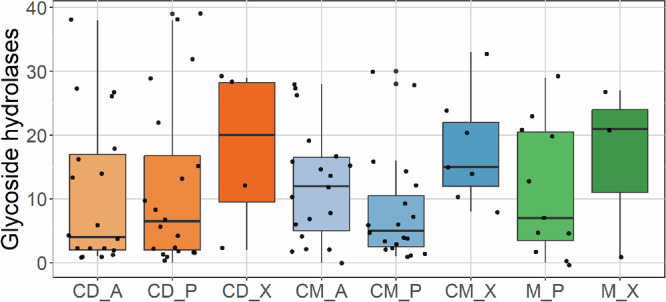
Boxplot with the count of genes encoding for glycoside hydrolases (EC 3.2.1.-) in the MAGs of the different enrichment cultures. CD compost and digestate, CM cow manure, M marshland soil, A Avicel®, P PASC, X xylan.

Regarding the metabolic potential of the enriched communities, we evaluated all enzyme classes according to the CAZYme classification. The two more enriched classes were glycosyltransferases and glycoside hydrolases, the latter being particularly more enriched in the cellulose-degrading cultures (Supplementary Fig. 12). Because each enrichment culture harboured different numbers of MAGs, we looked at the presence of genes encoding glycoside hydrolases (EC 3.2.1.-) in each genome. At least one gene was found in 98 of the 102 MAGs, and half of them have ten or more genes for enzymes of this family. Even though the cellulose enrichment cultures presented MAGs with a higher number of glycoside hydrolase genes, the xylan enrichment cultures had a higher median (Fig. 5).

**Fig. 6:**
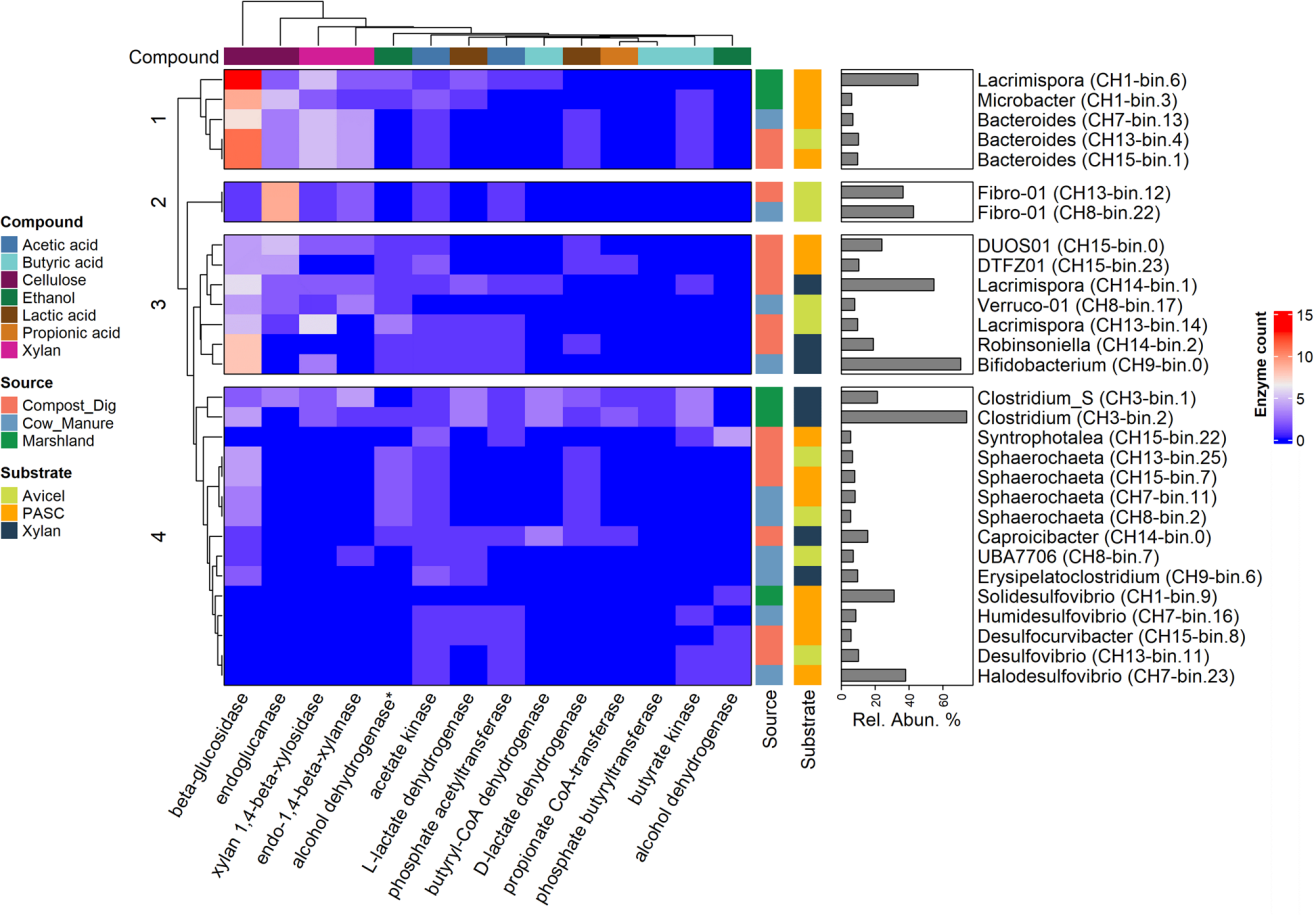
Heatmap constructed with the most abundant metagenome-assembled genomes (MAGs) (relative abundance >5%) recovered from the enrichment cultures. Gene counts for key enzymes from pathways for the degradation of cellulose and xylan or the production of acetic acid, butyric acid, ethanol, lactic acid, and propionic acid are shown. “Enzyme count” refers to the copy number of the corresponding gene in a particular MAG. Rel. Abun. – Relative abundance, Compost_Dig – compost and digestate. Fibro-01 belongs to the family Fibrobacteraceae. DUOS01 belongs to the family Treponemataceae. DTFZ01 belongs to the order Christensenellales. Verruc-01 belongs to the phylum Verrucomicrobiota. UBA7706 belongs to the class Bacilli.

In terms of the metabolic potential of the enriched communities, we focus in the following on the MAGs that had a relative abundance of >5% in each enrichment culture. In the clustered heatmap (Fig. 6), four different groups of MAGs can be observed. MAGs of groups 1 and 2 originate from different inoculum sources but were all enriched on cellulose, such as the *Bacteroides graminisolvens* MAGs CH7-bin.13 (cow manure), CH13-bin.4 and CH15-bin.1 (compost/digestate), and the Fibro-01 MAGs CH13-bin.12 (compost/digestate) and CH8-bin.22 (cow manure). For the Fibro-01 and *Bacteroides* MAGs, average nucleotide identities were higher than 97%, indicating that they represent one species of Fibrobacterota and *Bacteroides*, respectively. Concerning the production of carboxylic acids and ethanol, we found many genes of respective fermentation pathways in the MAGs, indicating their potential to produce such compounds, and they were spread in all four groups (Fig. 6).

### Metabolic modelling and community simulation

#### Community structures in simulations and substrate usage

GEMs were reconstructed from the MAGs for each enrichment culture, followed by community-based simulation of the metabolic phenotype. Most communities were characterised by a single highly abundant member with a metabolic profile not too different from other members, indicating dominance through competition rather than trophic organisation (Supplementary Figs. 13A–19A). Nonetheless, cross-feeding interactions were inferred, especially from the most to the least abundant members (Supplementary Figs. 13B-19B), via simple sugars, aldehydes/alcohols, and carboxylic acids. In some cases, mutualistic cross-feeding was predicted by the simulations, especially between the two most abundant community members (Supplementary Figs. 18 and 19), e.g., *Lacrimispora* and *Solidesulfovibrio* exchange mainly carboxylic acids as well as alcohols and aldehydes in the culture from marshland soil on PASC (Supplementary Fig. 18).

Experimentally high abundant taxa predicted to be involved in polysaccharide degradation also consumed oligosaccharides in the simulations. This refers to most of the communities, confirming *Lacrimispora* (Supplementary Fig. 18) and Fibro-01 (Supplementary Figs. 13 and 16) as key hydrolytic genera. Their MAGs also have high numbers of genes coding for polysaccharide degradation enzymes (Fig. 6).

#### Simulation of compound production

Comparing simulated production rates (Supplementary Fig. 20) with experimental results (Figs. 1–3), a good qualitative and quantitative agreement was observed. A significant positive correlation (Spearman’s *r* = 0.77, *p* < 0.01) was observed between simulated and experimental measurements (Supplementary Fig. 21). To better understand which community members contributed the most to the production of each compound, we clustered the simulated production profiles by genus (Supplementary Fig. 22). Based on the simulations, some members of *Firmicutes*, especially *Lacrimispora*, produced a broad range of compounds, which is consistent with the presence of fermentation-related genes (Fig. 6). Other community members showed a more specialised product spectrum, such as *Robinsoniella* and Verruc-01 that were the main propionic acid producers (Supplementary Fig. 22).

Several cases show alignment between the simulated community production (Supplementary Fig. 20), simulated production by genera (Supplementary Fig. 22), and experimental community production profile (Figs. 1–3). One case is *Caproicibacter*, which showed simulated butyric and caproic acid production (Supplementary Fig. 22). This genus was only observed in compost and digestate cultures on xylan - the sole community showing experimental production of caproic acid (Fig. 1). Another case is Verruc-01, which showed simulated propionic acid production (Supplementary Fig. 22). This genus was found in cow manure cultures on Avicel® and PASC, both of which showed experimental production of propionic acid (Fig. 2). *Fibrobacter* (Fibro-01) was a major player in the compost and digestate and cow manure cultures on Avicel®, and simulations indicate that it has a simple fermentation profile, contributing mostly to acetic acid and CO_2_ production in these communities (Supplementary Fig. 22). This is also consistent with its gene profile (Fig. 6).

#### Analysis of cross-feeding interactions

Using GEMs, we explored cross-feeding interactions unresolved by experiments. The most shared compounds (by total mass) were simple sugars (Supplementary Fig. 23), i.e., xylose and glucose, followed by fermentation products like acetaldehyde, diacetyl, glyceraldehyde, lactic acid, glycerol, and malic acid, reflecting the main carbon flow. Low-abundance compounds like nucleotides, amino acids, and vitamins were also cross-fed (Supplementary Fig. 24). Comparing metabolite consumption by abundance class (Supplementary Fig. 25) shows that highly abundant members are less likely to consume carboxylic acids, alcohols, and aldehydes compared to those with low abundance (Mann-Whitney U test, *p*-value < 0.05).

### Case-study: xylan enrichment culture from compost and digestate

**Fig. 7:**
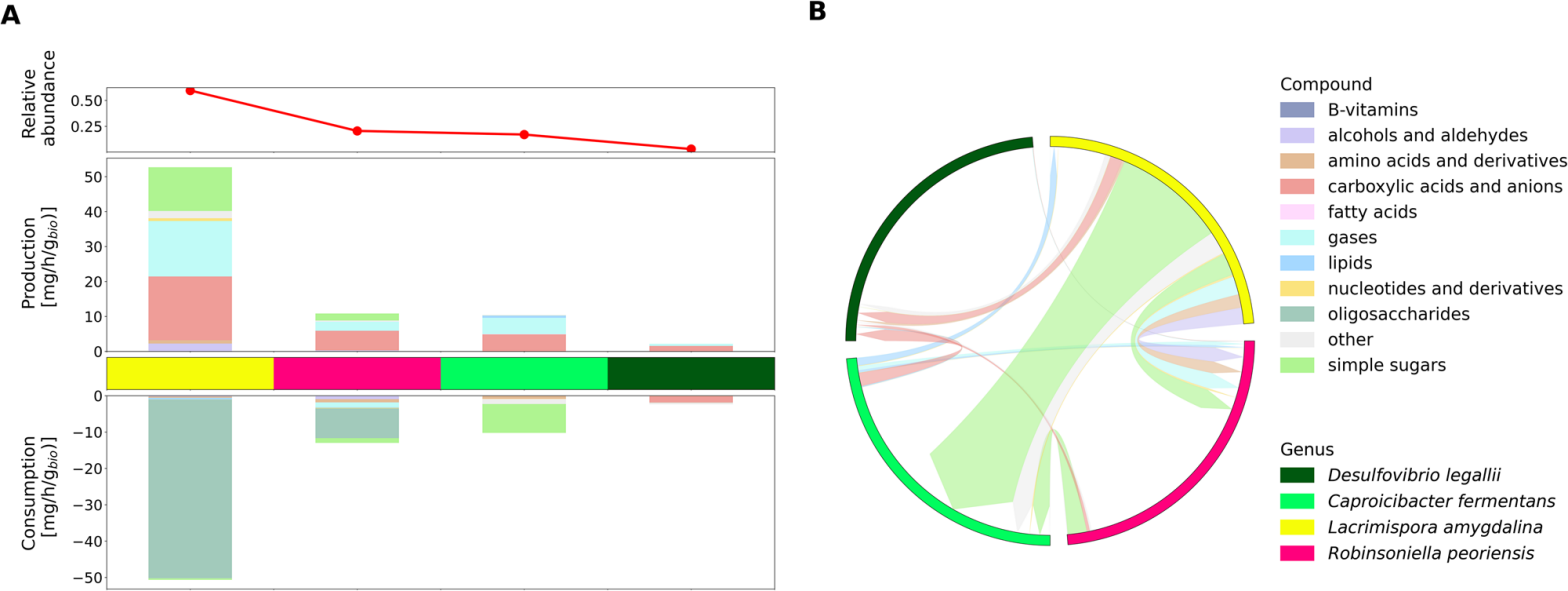
Simulation results for the xylan enrichment culture from compost and digestate. **A** Relative abundance as well as consumption and production profiles of each community member, including the total amount of each compound class released and consumed from the growth medium and the amount shared with other members; **B** cross-feeding interactions between community members (arrow thickness is proportional to mass rate). All results represent average flux distributions from 1000 randomly sampled solutions and are presented in rates of total mass exchanged (mg of compound per g of community dry weight per hour).

Due to the high product diversity in the compost and digestate enrichment on xylan, we analysed this community in detail by modelling and integrating the results with functional annotations derived from metagenomic data, to generate hypotheses on potential metabolic interactions that could not be resolved from experimental metabolite measurements alone. The community consisted of four species with uneven abundance distribution: *Lacrimispora amygdalina* CH14.1 (60%), *Robinsoniella peoriensis* CH14.2 (20%), *Caproicibacter fermentans* CH14.0 (17%), and *Desulfovibrio legallii* CH14.4 (3%). Model simulations revealed that metabolite exchange correlated with abundance, with dominant species exchanging compounds with higher mass rate (Fig. 7A). Simulations further suggested that all species primarily produced acetic acid and CO_2_; however, distinct metabolic roles were inferred from the model outputs. In particular, *C. fermentans* was predicted to be the main producer of butyric acid and the sole producer of caproic acid, while *L. amygdalina* and *R. peoriensis* were predicted to degrade xylan, releasing xylose that could serve as a substrate for potential cross-feeding partners (Fig. 7B). *C. fermentans* was additionally predicted to produce hydrogen, and receive glycerol and glyceraldehyde from *R. peoriensis* and *L. amygdalina*. The minor community member *D. legallii* was predicted to act as a sink for fermentation products, such as carboxylic acids, without contributing to xylan degradation.

**Fig. 8:**
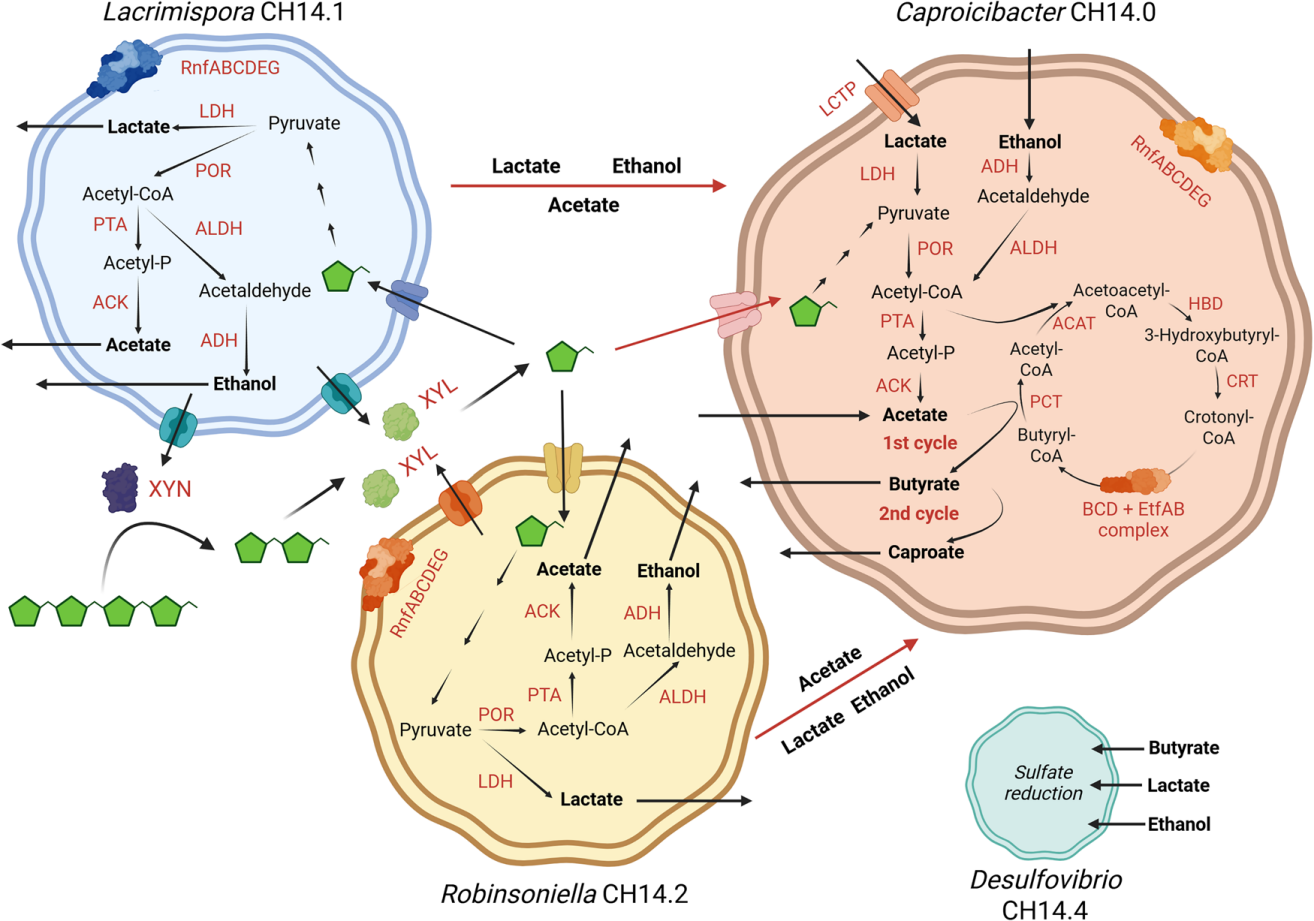
Trophic network and metabolic fluxes within the community model of the xylan enrichment culture derived from compost and digestate growing on xylan, based on metagenome annotations. Red arrows indicate metabolite exchange between community members, with the exception of *Desulfovibrio legallii* (CH14.4), which, due to its low relative abundance, is likely less involved but may utilise metabolic by-products. Black arrows with increasing thickness indicate the preferred direction of enzyme reaction. The enzymes of dissimilatory sulphate reduction in CH14.4, as well as those involved in xylose degradation and subsequent glycolysis (multi-step arrows from xylose to pyruvate) in CH14.0, 14.1, and 14.2, are not listed individually; however, all corresponding genes were found (Supplementary Dataset 6). Black arrows of uniform thickness across membranes indicate uptake and efflux processes, either through diffusion or transporter-mediated. Transporter symbols represent transporter-mediated transport processes. Xylose molecules are shown as green symbols, which, when linked represent xylobiose and xylan. Enzyme abbreviations are shown in red: XYN endo-1,4-beta-xylanase, XYL xylan 1,4-beta-xylosidase, XYLA-II xylA-II, XYLB xylulokinase, TKT transketolase, POR pyruvate-ferredoxin/flavodoxin oxidoreductase, PTA phosphate acetyltransferase, ACKA: acetate kinase, ADH alcohol dehydrogenase, LDH L-lactate dehydrogenase, LCTP lactate permease, ACAT acetyl-CoA C-acetyltransferase, HBD 3-hydroxybutyryl-CoA dehydrogenase, CRT enoyl-CoA hydratase, BCD butyryl-CoA dehydrogenase, EtfA/B electron transfer flavoprotein alpha and beta subunit, PCT propionate CoA-transferase, RnfABCDEG Rnf electron transport complex, PTB phosphate butyryltransferase. Corresponding EC numbers and further information for each enzyme can be found in Supplementary Dataset 6. All acids are presented in their salt forms. Recovered MAGs: *Lacrimispora amygdalina* (CH14.1)*, Caproicibacter fermentans* (CH14.0), *Robinsoniella peoriensis* (CH14.2), and *Desulfovibrio legallii* (CH14.4).

Metagenomic annotations supported these predicted roles by revealing key enzymes involved in xylan hydrolysis and xylose metabolism (Fig. 8; Supplementary Dataset 6). *L. amygdalina* encodes both endo-1,4-beta-xylanase (XYN) and xylan 1,4-beta-xylosidase (XYL), enabling extracellular breakdown of xylan to xylobiose and further to D-xylose. *R. peoriensis* also encodes XYL activity, reinforcing its predicted role in xylan hydrolysis. The three most abundant community members *L. amygdalina*, *R. peoriensis*, and *C. fermentans* harbour the full pathway for intracellular xylose catabolism via XYLA-II^16^, XYLB, TKT, and the glycolytic conversion (Supplementary Dataset 6) to pyruvate. *L. amygdalina* and *R. peoriensis* further encode enzymes for the production of acetic acid, ethanol, and L-lactic acid, while *C. fermentans* was predicted to utilise these fermentation products via reverse β-oxidation to produce butyric and caproic acid. In this trophic network, *D. legallii* acts likely as a product scavenger of butyric acid, lactic acid and ethanol.

## Discussion

The high potential of systematic analyses of microbial communities via metagenomics, gene annotation, metabolic reconstruction and community simulations has been reviewed recently^17^, and similar approaches have been applied to lignocellulose-enriched communities^18–20^. Previous studies have also demonstrated GEM reconstruction and community simulations from MAGs^21^. In this study, we extended these approaches by combining MAG-based pathway reconstruction with simulation-supported modelling of trophic interactions across multiple substrates and inocula within a unified experimental framework. A distinguishing aspect of our work is the integration of experimentally informed manual curation of the metabolic models and community simulations using an updated implementation of SteadierCom. This comparative approach enabled the analysis of substrate- and inoculum-dependent product profiles and the reconstruction of functionally differentiated trophic networks. Overall, these results provide a framework for the functional analysis and prediction of microbial interactions in lignocellulose-based systems.

The enrichment process selectively reduced community complexity while enhancing and stabilising desired metabolic functions. It should be noted that the use of different pH conditions limits the direct comparability of the enriched microbial communities, since pH strongly influences microbial selection and metabolic activity. However, the different pH values were intentionally chosen to reflect the native conditions of the inocula and to investigate enrichment performance under conditions relevant to microbial chain elongation and carboxylate production systems, which are commonly operated across a range from mildly acidic to neutral pH values.

The substrate-specific minimal media exerted a selective pressure towards the growth of primary degraders (trophic level 1) and secondary consumers (trophic level 2 or higher). These trophic levels and interactions were resolved in detail in the xylan enrichment from compost and digestate, the only culture that experimentally produced caproic acid. This analysis extends previous model-based studies of microbial interactions^22^, and represents the first MAG-based pathway and dynamic trophic network reconstruction of a xylan-enriched community under defined conditions. According to our community model, *Lacrimispora amygdalina* and *Robinsoniella peoriensis* provided acetic acid, ethanol, and lactic acid to *Caproicibacter fermentans*, although simulations predicted that *C. fermentans* would receive simple sugars and compounds other than carboxylic acids and alcohols. Nonetheless, the observed caproic acid formation and predicted dominance in butyric acid production by *C. fermentans* were consistent with model outcomes. Interestingly, ethanol was detected experimentally while lactic acid was not, implying that *C. fermentans* preferred lactic acid as electron donor for chain elongation. Moreover, MAGs assigned to *R. peoriensis* revealed so far uncharacterised metabolic capabilities, including lactic acid, acetic acid and ethanol production as well as the ability to degrade xylobiose and utilise xylose, which expands the currently limited knowledge of this species’ ecological and metabolic potential.

The genus *Robinsoniella* played also an important role in the enrichment with cow manure on xylan, in which we observed lactic acid production. Based on the simulation results, most members, including *Robinsoniella* and *Bifidobacterium*, were consuming simple sugars. Also, in this culture, *Robinsoniella* seemed to promote the production of lactic acid, acetic acid and ethanol. *Bifidobacterium* is a well-known lactic acid producer^23^. To our knowledge, this is the first documented anaerobic xylan enrichment in which lactic acid is the dominant fermentation product, consistently reaching concentrations above 1 g l^-1^. Lactic acid serves not only as an electron donor for microbial chain elongation^14^, but also represents a valuable platform chemical with industrial relevance, such as in the synthesis of polylactic acid^24^.

Enrichments from marshland soil on cellulose yielded the lowest biomass and product concentrations, with poor DNA recovery preventing sequencing in some samples. The detected genera corresponded to taxa commonly found in marshlands^25,26^, but showed limited product formation in our study. In contrast, the marshland soil enrichment grown on xylan showed higher product concentrations. MAGs assigned to *Clostridium* indicated potential for xylan degradation and organic acid production, consistent with the known role of this genus with butyric acid production^27^ associated with hydrogen production^28,29^. Although the concentrations reached in this study were lower than those achieved in industrial processes, the results highlight the potential of such communities and expand the known substrate spectrum for butyric acid fermentation. These findings provide insights into this specific marsh ecosystem but cannot be generalised to the diverse range of wetland types. Given that many wetlands harbour active anaerobic fermentation, further studies are needed to explore their biotechnological potential.

Based on the simulations, *Fibrobacter* and *Lacrimispora* emerged in many cellulose enrichments to be the primary degraders. *Fibrobacter* is known to break down cellulosic plant biomass within the gut of herbivores^30^. The two *Fibrobacter* MAGs were highly similar (98.9% average nucleotide identity), even though they came from different inoculum sources, and harbour several copies of endoglucanase genes. This suggests that the selective pressure with microcrystalline cellulose as sole carbon source favoured one species of this genus. Representatives of *Lacrimispora* were present in several communities, both with xylan and cellulose as the carbon source. From the genus *Lacrimispora* cellulose as well as xylan degrading strains are known^31–33^. In the cellulose cultures, simulations indicate that *Lacrimispora* produced a broad range of fermentation products, underscoring the metabolic flexibility of Lachnospiraceae^34^. Their MAGs also harbour genes associated with the degradation of cellulose and hemicellulose. The simulations also suggest that *Lacrimispora* consumed carboxylic acids and, in some cases, directly contributed to the production of caproic acid in cellulose-grown communities, although this was not experimentally confirmed.

In addition to the dominant taxa in cellulose-enriched communities previously discussed, *Bacteroides* and *Sphaerochaeta* were detected with substantial abundance and may fulfil distinct ecological roles. *Bacteroides*, particularly MAGs affiliated with *B. graminisolvens*, was abundant in four out of the six cellulose cultures. While the type strain of this species is known to utilise cellobiose but not cellulose^35^, our MAGs encoded multiple copies of endoglucanase and β-glucosidase genes. However, simulations suggested limited uptake of oligosaccharides, indicating a preference for smaller hydrolysis products such as glucose and cellobiose. The recurring presence of *Bacteroides* across different enrichments suggests thus a functional role as a secondary degrader. *Sphaerochaeta* was identified as a potential contributor to propionic acid formation, a product observed in several cellulose cultures. Although MAGs displayed the genomic potential to degrade cellulose and hemicellulose, simulation results indicated only limited engagement in primary degradation pathways. Instead, their metabolic activity may be linked to the fermentation of intermediate metabolites, positioning them as secondary fermenters within the trophic network. *Sphaerochaeta* belongs to the phylum Spirochaetota, which has often been observed in anaerobic cellulolytic communities. It can be hypothesised that they kept the cellobiose concentrations low and this way prevented the inhibition of cellulase systems of the cellulolytic bacteria^36,37^.

*Desulfovibrio* was detected at high relative abundance in several cellulose enrichments and at lower abundance in the xylan-grown community from compost and digestate. Members of this genus are known for dissimilatory sulphate reduction, coupling the oxidation of electron donors, such as ethanol, lactic acid, or hydrogen to sulphate as terminal electron acceptor^38^. Simulations suggested that *Desulfovibrio* occasionally consumed oligosaccharides, a finding that contradicts known metabolic traits of this genus and likely reflects model limitations due to misannotations or gap-filling (see Supplementary Note 4.2 and Supplementary Fig. 26). Nonetheless, genomic analysis indicated the presence of genes encoding key enzymes for lactic acid and butyric acid utilisation, including lactate permease, lactate racemase, phosphate butyryltransferase (PTB), and butyrate kinase (BUK). Lactate dehydrogenase (LDH) genes were not recovered, which may be due to incomplete MAGs.

In the compost and digestate xylan community, *Desulfovibrio* may compete with chain elongators such as *Caproicibacter* for lactic acid and ethanol. However, its ecological impact appears limited due to low sulphate concentrations in the medium, which likely restricted its growth. Its ability to utilise hydrogen from fermentation or chain elongation reactions further supports its role as a flexible, though low-abundant, product scavenger in the anaerobic network. Importantly, sulphate reduction by *Desulfovibrio* can influence system performance beyond metabolic competition. Sulphide production may inhibit sensitive microbial populations and precipitate trace metals, reducing their bioavailability^39,40^. On the other hand, the precipitation effect may offer benefits for bioprocesses involving lignocellulosic waste with elevated heavy metal content^40^, such as residues from the pulp and paper industry^41–43^. These residues, rich in cellulose and hemicellulose^44,45^, are promising feedstocks for microbial valorisation, and the resilience of our enrichment cultures to such inhibitory compounds may aid in improving their biotechnological utility.

Some community members (e.g., *Solidesulfovibrio*, *Humindesulfovibrio*, *Desulfovibrio*) were predicted to exhibit reverse metabolic activities, including potential production of electron donors such as L-lactic acid. These predictions were derived from GEMs that required gap-filling to achieve metabolic feasibility and may therefore reflect uncertainties in pathway directionality or annotation quality. Experimentally, metabolite profiles were determined using HPLC, which does not directly resolve the direction of metabolite exchange between individual community members. Consequently, inferred cross-feeding interactions (e.g., potential lactate transfer between community members including *Lacrimispora*) are model-derived hypotheses rather than experimentally validated interactions. Overall, while the integrated models capture the observed spectrum of metabolites produced by the communities, quantitative agreement is limited, and predicted flux distributions should be interpreted with caution due to inherent uncertainties in gap-filling procedures and the non-uniqueness of feasible flux solutions in genome-scale metabolic models. Future work could reduce these uncertainties by incorporating additional constraints, such as gene expression data, compound toxicity effects, or time-resolved dynamic modelling approaches such as multi-species dynamic flux balance analysis^46^.

It should be considered that this study used model substrates instead of straw, which may limit the ecological significance and practical applicability of the findings. The model substrates lacked lignin and the structural complexity of natural lignocellulosic materials, where cellulose, hemicellulose, and lignin are closely interconnected. Consequently, the substrates used here were more accessible for microbial degradation than straw. In the presence of more complex lignocellulosic substrates, such as straw, the community composition and metabolic interactions may differ. In particular, spatial heterogeneity in natural substrates can create more ecological niches and alter cross-feeding interactions^47,48^. Nevertheless, the general trophic organisation observed in this study, including primary degraders, secondary consumers, and non-degraders linked through cross-feeding, may also be found during degradation of more complex substrates, as similar functional guilds emerged reproducibly across different enrichment conditions. Thus, our investigation provides an initial framework for understanding microbial interactions during lignocellulose degradation, while future studies should assess the transferability of these findings to straw and other complex lignocellulosic substrates.

Altogether, we identified degraders such as *Lacrimispora* and *Fibro-01* that occurred across substrates and inocula (Supplementary Fig. 27). *Bacteroides* and *Sphaerochaeta* formed a key guild of secondary consumers, abundant in nearly all cellulose enrichments (Supplementary Fig. 27), which probably mainly produced acetate. Xylan enrichments exhibited broader product spectra. The secondary consumer *Robinsoniella* was present in two of three xylan enrichment cultures. Secondary consumers utilise degraders’ products and in return supply vitamins, cofactors, and amino acids, while avoiding direct competition through specialisation. This division of labour enhances consortium performance, increases ecological flexibility, and supports resilience^49^. Terminal oxidisers such as *Desulfovibrio* can stabilise lignocellulose degrading communities by consuming H_2_, which shifts reaction equilibria, supports product accumulation, and promotes secondary fermentations^50^. These functional roles of degraders, secondary consumers, and terminal oxidisers, consistently emerged in our cultures irrespective of substrate or inoculum, similar as observed in the human gut microbiome^51^.

Based on this modular principle, synthetic consortia could be designed for the targeted production of bio-chemicals. The choice of genera for each functional group can be based on those that repeatedly appeared in our system, as their consistent presence across different substrates and inocula demonstrates they are robust candidates. Such synthetic consortia could bring several advantages over complex mixed cultures, including improved process control, reduced formation of unwanted by-products, higher reproducibility, and the possibility to tailor the community composition towards specific metabolic functions and target products. However, the transfer of these systems to industrial bioprocesses remains challenging. Since this study was conducted exclusively in small-scale batch cultures, important parameters for process design, such as the growth kinetics and long-term stability of individual consortium members, remain unknown. In continuous systems, differences in growth rates could alter the community composition and affect process performance. In addition, non-sterile lignocellulosic feedstocks may introduce competing microorganisms that could displace members of the synthetic consortium. Therefore, such consortia may be particularly suitable for low-contamination lignocellulosic substrates or pretreated feedstocks, such as steam-exploded straw, hydrolysates, or paper waste. Maintaining sterile or highly controlled conditions could improve community stability, but would also increase process complexity and costs. A logical next step would therefore be to investigate promising synthetic consortia or selected enrichment cultures in controlled bioreactor systems under process conditions more relevant to industrial applications.

## Methods

### Experimental design of the enrichment process

Enrichment was done with four inocula (marshland soil, cow manure, and compost combined with digestate), selected to represent environmentally relevant sources of lignocellulose-degrading microbial communities with differing ecological backgrounds. Marshland soil is a potential source of anaerobic lignocellulose-degrading bacteria^52,53^. It was collected in the Presseler Heide nature reserve, Germany (51°34’35.6” N; 12°45’29.8” E) in December 2021. The samples were taken with a sediment auger at a depth of at least 30 cm, and had a pH value of 4.2. Cow manure was included due to the presence of rumen-derived microbial communities specialised in plant biomass degradation^54^. The cow manure sample (volatile organic acids (VOA): 8.22 g l^−1^, pH 7.18) was obtained from a cesspit of a dairy farm close to Leipzig, Germany, in November 2021. Compost and digestate were selected as representatives of engineered microbial ecosystems with high functional diversity and strong lignocellulose-degrading potential^55^. The compost sample (pH 7.59) was taken from a compost heap fed exclusively with plant material from a private garden near Leipzig in December 2021. Digestate was sampled from a lab-scale biogas reactor fed with grain husks (process parameters at sampling: pH: 7.51, VOA: 0.68 g l^-1^, VOA/total inorganic carbon: 0.13 gVOA/gCaCO_3_, ammonia nitrogen content (NH_4_-N): 1.88 g l^-1^, total solids (TS): 2.87%, volatile solids (VS): 78.6%TS, 39 °C, hydraulic retention time: 38.6 d, organic loading rate: 2.5 gVS l^-1^ d^-1^). The samples of each inoculum were stored in bottles that had been flushed with nitrogen before. The bottles were completely filled, sealed with airtight lids and stored at 4 °C until the beginning of the experiment.

Cultivation was done anaerobically in 100 ml mineral medium SynCon1, which was designed for the enrichment (Supplementary Dataset 7). The medium contained a vitamin solution, either a Gomori buffer to maintain a neutral pH or 2-(*N*-morpholino)ethanesulphonic acid buffer for acidic pH, and trace element solution SL-10 as described for DSMZ medium 320 (https://mediadive.dsmz.de/medium/320). Three different carbon sources were used at 5 g l^-1^, being xylan (≥95% xylooligosaccharides from maize cob; Carl Roth, cat. 8659.3), phosphoric acid swollen cellulose (PASC), or Avicel® (microcrystalline cellulose; Sigma-Aldrich, cat. 11363). PASC was prepared from Avicel® according to the method described by Franche et al.^56^. In order to remove traces of oxygen, the medium was stirred for 30 min in an anaerobic chamber with an atmosphere of 98% N_2_ and 2% H_2_. It was then gassed with N_2_ for 10 min at 1000 l min^−1^ to remove H_2_, and autoclaved (20 min, 121 °C). The pH was adjusted with 1 M HCl or 1 M NaOH.

Initially, anaerobic 200-ml serum bottles containing the medium (100 ml) and one of the carbon sources were inoculated with 1 g of inoculum in the anaerobic chamber and closed with butyl septa and aluminium crimp caps. For the bottles with compost and digestate, 0.5 g of each inoculum was added. For each inoculum and carbon source combination, three bottles were prepared, consisting of two biological replicates and one negative control. Thus, each transfer comprised a total of 27 bottles. The cultures were incubated at 33 °C and transferred every seven to 15 days to fresh medium (1:20 dilution). Shorter initial intervals were used to enrich fast-growing degraders, while longer intervals later in the enrichment allowed for more complete substrate conversion and the development of cross-feeding interactions. The exact cultivation duration of each transfer number can be found in Supplementary Dataset 8. If methane production was observed during the enrichment, 2-bromoethanesulfonate was added at a final concentration of 12 g l^-1^ in the following two transfers. The pH was adjusted to 5.5 ± 0.5 for the enrichment cultures with marshland soil as inoculum and to 7.2 ± 0.5 for the other cultures, in order to approximate the initially measured pH values of the respective source environments. The pH for marshland soil represents a compromise between the native acidic conditions and conditions that allow efficient microbial chain elongation and metabolite formation. The pH was measured with a benchtop pH metre (LAQUAtwin pH Tester; Horiba) and adjusted, if necessary, several times per week up to transfer number 3 (cultures with PASC and marshland soil), 4 (cultures with PASC and cow manure or compost and digestate) or 5 (all other cultures), and thereafter once a week. If the pressure in the bottles reached 0.4 bar(g), it was reduced through degassing. Negative control bottles were inoculated but did not contain cellulose or xylan. These controls were included to assess whether microorganisms were enriched on alternative carbon sources present in the medium or inoculum rather than on cellulose or xylan.

For the enrichment cultures from cow manure and marshland soil with PASC and Avicel®, we observed only a low production performance from transfer number 10 on. Therefore, the experiment was repeated using transfer number 4 as inoculum with a modified mineral medium, SynCon2 (Supplementary Dataset 7), in parallel to the ongoing experiment with SynCon1. Compared to SynCon1, SynCon2 did not contain ascorbic acid, but Na_2_SeO_3_ and Na_2_WO_4_ to provide selenium and tungsten as additional trace elements.

### Process analytics

The formation of organic acids and alcohols in the enrichment cultures was monitored using high-performance liquid chromatography (HPLC) based on the method of Afzal et al.^57^ Analyses were performed using an HPLC with a refractive index detector, a PL Hi-Plex H guard column, and a Hi-Plex H analytical column. Liquid products of the marshland soil cultures were analysed using headspace gas chromatography (GC) according to Apelt^58^, since the 2-(*N*-morpholino)ethanesulphonic acid buffer coeluted with propionic acid in our HPLC. The protein concentration was determined with a modified Bradford protein assay^59^. Headspace composition of the cultures was analysed by GC (Micro GC CP-2002P, Chrompack) using a Molsieve column (5 A PLOT, 30 m length, 0.53 mm diameter) with an Rt-Q-Bond pre-column (3 m length) separating H_2_, N_2_, O_2_ and CH_4_, and an Rt-Q-Bond column (12 m length) separating CO_2_.

The most efficient enrichment cultures were analysed regarding their capability of degrading xylan or Avicel®. The enrichment cultures were cultivated for either two (xylan enrichment cultures) or five weeks (Avicel® enrichment cultures) after completion of the enrichment process. The cultivation bottles were then opened and samples were taken while stirring the culture to ensure homogeneous samples. The xylan or cellulose content was measured based on the modified phenol-sulphuric acid method described by Hemme et al.^60^. For each enrichment culture, two biological replicates and a substrate-free control, which was also inoculated, and sterile controls were analysed. Sterile controls were treated in the same way as the enrichment cultures, the xylan and cellulose contents were then determined and the values were considered as starting values.

Sample preparation, operating conditions, and specifications about the instrumental settings for each method are described in the Supplementary Information.

Plots to visualise the output of the process analytics were created using R v.4.3.0 (package: tidyverse). Code refinement was supported by ChatGPT-4o, limited strictly to improving self-written code, without generating content, or processing data.

### Microbial community analysis

DNA from 2-ml enrichment culture samples was extracted using the NucleoSpin® Tissue DNA kit (Macherey-Nagel) according to the manual with minor modifications. Samples were incubated for five hours in the pre-lysis sample step, followed by the recommended protocol until the DNA elution step, where we proceeded with the recommendations of the manual to increase yield and concentration. DNA was quantified using the Qubit™ dsDNA BR Assay Kit (Invitrogen) and concentrated using Amicon® centrifugal filters (Merck) when the concentration was below 5 ng µl^-1^.

Microbial community composition was assessed via 16S rRNA gene amplicon sequencing (220 samples) and shotgun metagenomics (eight samples – last time point of the enrichment cultures). Amplicons were generated using the primer set 341 f (5’-CCT ACG GGN GGC WGC AG-3’) and 785r (5’-GAC TAC HVG GGT ATC TAA TCC-3’), which targets the V3-V4 region^61^ of the 16S rRNA gene. A library was prepared according to the Illumina manual and sequenced on the MiSeq platform using the MiSeq Reagent Kit v3 with 2× 300 cycles (Illumina). The DADA2 pipeline v.1.28.0^62^ in R v.4.3.0^63^ was used to infer amplicon sequence variants (ASVs), and phyloseq v.1.44.0^62,64^ to perform the compositional analysis. The database used for taxonomic assignment was Silva v.138.1^65^.

Shotgun metagenome analysis was performed with stable enrichment communities, i.e., only the second to last and/or the last transfers of every enrichment culture were used. Sequencing for shotgun metagenome analysis was performed using the Illumina NovaSeq system (2 × 150 bp) by Azenta Life Sciences. MAGs were recovered with the MuDoGeR pipeline (v.1.0.1)^66^. MetaSpades v.3.15.5^67^ was used for the assembly and metaWRAP v.1.3.2^68^ for the binning process. Quality and taxonomy were assessed with CheckM v.1.0.18^69^ and GTDB-tk v.2.1.1 release 214^70^, respectively. Functional annotation of high-quality MAGs (completeness >90% and contamination <5%) was performed with DRAM v.1.4.6^71^. Annotation of genes encoding important metabolic functions was manually curated (Supplementary Dataset 9), and BlastKOALA v.3.1^72^ was used to curate metabolic pathways. CoverM v.0.7.0^73^ was used to calculate the MAGs’ relative abundance. Proksee Map Builder v.2.0.5^74^ was used for genome visualisation. The average nucleotide identity was calculated with the FastANI software v.1.1.1^75^ available via Proksee. SignalP v.5.0 was used to search for signal peptides of particular enzymes^72^.

Alpha-diversity indices Shannon and Simpson, and non-metric multi-dimensional scaling (NMDS) ordination based on the Bray-Curtis distance were calculated with phyloseq v.1.44.0^62,64^. PERMANOVA tests were calculated using vegn v.2.6.4^76^. Spearman’s correlation analysis was performed using the R stats package^63^, after normalising the amplicon data with centred log-ratio function from the package composition^77^.

### Metabolic modelling

#### Model building

To reconstruct GEMs from the MAGs, we used CarveMe v.1.6.1^78^ and Gurobi v.11.0.1 (Gurobi Optimization, LLC). To account for pathways absent in CarveMe’s universal model but essential for our system, such as reverse β-oxidation and polysaccharide degradation, we integrated curated reactions and metabolites from previously reconstructed models^79,80^. Preliminary analysis of the universal metabolic model used by CarveMe as a basis for reconstruction revealed missing pathways crucial for our enrichment cultures, including MCCA production through the reverse β-oxidation pathway and degradation pathways for various polysaccharides. We have previously reconstructed the reverse β-oxidation pathway for two-chain elongators based on the results from Liu et al.^79^, and we have reconstructed and curated a model for a polysaccharide degrader^80^, which contains the missing reactions and metabolites. Therefore, these reactions and metabolites were merged into the universal model prior to reconstruction.

To ensure that each community member was able to grow on a combination of the nutrients provided in the synthetic medium and the compounds provided by other community members, we used a two-step gap-filling approach. First, reconstructions were performed using a rich anaerobic medium (LB composition in CarveMe). Flux variability analysis^81^ was then used to determine the production ability of each member. In a second round, each community member was reconstructed, using as growth medium a combination of SynCon1 or SynCon 2 medium with the compounds produced by other community members. In addition, we used the soft-constraint feature in CarveMe to prioritise secretion of the compounds present in the HPLC results.

#### Community simulation

Simulation of the metabolic phenotype of each enrichment culture was performed using SteadierCom^82^, an extension of the community simulation method SteadyCom^83^ that incorporates balanced community growth (i.e., all members have the same specific, but not absolute, growth rate), constraints on maximal enzyme capacity, relative species abundance, and total community growth rate. The use of a family-level taxonomic resolution is based on previously reported observations of conservation of metabolic function at family level^84^. We used the dilution factor and divided it by the time between the serial transfers to estimate the average growth rate in the final stages of enrichment. The relative abundances were estimated from the shotgun sequencing data. The simulations were performed using SynCon1 or SynCon2 growth medium with cellulose or xylan oligosaccharides as carbon source, respectively. We performed random sampling to obtain a set of 100 alternative solutions for each community. For each simulation, SteadierCom outputs the frequency and the mean flux of each potential cross-feeding interaction detected across the sampled solutions.

#### Post-simulation analysis

Barnard’s exact test^85^ and Benjamini–Hochberg correction^86^ were used to study statistical associations between families and exchanged compounds across our enriched consortia. We also used the one-sided Mann-Whitney U-test for studying difference in dependence on different classes of compounds between members of low abundance (<10% of community) and high abundance (≥10% of community). The tests were executed with the stats module in SciPy v.1.7.1^87^. Auxotrophies were studied for individual models for members of the compost and digestate community on xylan, using Reframed v.1.5.2^88^. In addition, we performed a correlation analysis to assess the agreement between model-predicted metabolite production and experimentally measured metabolite concentrations.

## Supplementary information

Supplementary information

Supplementary Dataset 1

Supplementary Dataset 2

Supplementary Dataset 3

Supplementary Dataset 4

Supplementary Dataset 5

Supplementary Dataset 6

Supplementary Dataset 7

Supplementary Dataset 8

Supplementary Dataset 9

## Data Availability

Sequence data are available under NCBI BioProject ID PRJNA1177406. The data supporting the findings are included in the Supplementary Information and Datasets.
