# Supplementary material for: Functional roles of degraders and non-degraders in anaerobic trophic networks converting lignocellulose into monocarboxylates
Source: NPJ Biofilms Microbiomes. 2026 Jun 24;12:126. doi: 10.1038/s41522-026-01072-x (PMC13294492; doi:10.1038/s41522-026-01072-x)
Supplement: Supplementary file 1 — Supplementary information [file 41522_2026_1072_MOESM1_ESM.pdf]

Supplementary Information for the manuscript:

Functional roles of degraders and non-degraders in anaerobic trophic networks converting lignocellulose into monocarboxylates

Christina Schäfer[1], Maria L. Bonatelli[1,2], Idun Maria Tokvam Burgos[3], Sabine Kleinsteuber[1], Daniel Machado[3], Ove Øyås[4], Hauke Harms[5], Heike Sträuber[1]*

1 Helmholtz Centre for Environmental Research – UFZ, Department of Microbial Biotechnology, Leipzig, Germany

2 Martin Luther University Halle-Wittenberg, Department of Genetics, Halle, Germany

3 Norwegian University of Science and Technology (NTNU), Department of Biotechnology and Food Science Trondheim, Norway

4 Oslo Centre for Epidemiology and Biostatistics, University of Oslo and Oslo University Hospital, Oslo, Norway

5 Helmholtz Centre for Environmental Research – UFZ, Department of Applied Microbial Ecology, Leipzig, Germany

* corresponding author, e-mail: heike.straeuber@ufz.de

Table of content

1. Methods 4
1.1 Analysis of liquid products 4
1.2 Modified Bradford protein assay 4
1.3 Sampling for gas composition analysis, and data analysis 4
1.4 Modified phenol-sulphuric acid method 5
2. Production performance of enrichment cultures 5
2.1 Controls 5
Figure 1 6
Figure 2 7
2.2 Relative gas composition of enrichment cultures from compost and digestate 8
Figure 3 8
2.3 Relative gas composition of enrichment cultures from cow manure 9
Figure 4 9
2.4 Relative gas composition of the enrichment cultures from marshland soil 10
Figure 5 10
2.5 Fermentation products and protein concentrations of cellulose enrichment cultures 11
Figure 6 11
2.6 Degradation of xylan or Avicel® in selected enrichment cultures 12
Figure 7 12
3. Microbial community analysis 13
3.1 Spearman's correlation analysis 13
Figure 8 13
3.2 Alpha- and beta-diversity analysis 14
Figure 9 14
Figure 10 14
3.3 Community composition from amplicon sequencing data at phylum level 15
Figure 11 15
Figure 12 16
4. Metabolic modelling 17
4.1 Simulation results 17
Figure 13 17
Figure 14 18
Figure 15 19

Figure 16 20
Figure 17 21
Figure 18 22
Figure 19 23
Figure 20 24
Figure 21 25
Figure 22 26
Figure 23 27
Figure 24 28
Figure 25 29
Figure 26 30
Figure 27 31
4.2 Limitations and contradicting results 31
5. References 32

1. Methods

1.1 Analysis of liquid products

For HPLC analysis, liquid samples of the cultures were centrifuged at 6,000 × *g* for 10 min at 4 °C. Supernatants were collected and filtered (0.22 µm, cellulose acetate). The analyses were performed using an HPLC (Shimadzu) with a pre-column PL Hi-Plex H Guard (50 mm length, 7.7 mm diameter; Agilent Technologies) and a Hi-Plex H column (300 mm length, 7.7 mm diameter; Agilent Technologies). The operating conditions were: oven temperature 55 °C, injection volume of 20 µl, 5 mM sulphuric acid as eluent (isocratic), flow rate of 0.7 ml min^{-1}, injector temperature of 90 °C, TCD temperature of 70 °C, TSCD Delta temperature of 20 °C, sample inlet heater temperature of 70 °C, backflush time of 9 s, and sample pump time of 25 s. Formic acid, acetic acid, propionic acid, lactic acid, isobutyric acid, butyric acid, isovaleric acid, valeric acid, isocaproic acid, caproic acid, ethanol, 1-propanol and 1-butanol were detected using a refractive index detector (Shimadzu).

For headspace GC analysis, 3 ml sample, 1 ml control standard (2-methylbutyric acid, 184 mg l^{-1}), 0.5 ml methanol and 2.5 ml 78.4% sulphuric acid were given in a 20-ml GC vial, and the vial was sealed with an aluminium crimp cap with PTFE/silicone septum. The samples were stored at 4 °C until measurement. The samples were analysed with a 7890 A gas chromatograph with flame ionisation detector (260 °C; Agilent Technologies) and autosampler (Turbomatrix110; Perkin Elmer). Separation was done by a DB-FFAP column (60 m × 0.25 mm × 0.5 µm; Agilent Technologies). The injection mode was set to a split ratio of 1:10, with the injector temperature maintained at 220 °C. The gas chromatography system operated in programmed-temperature mode as follows: an initial temperature of 40 °C held for 20 minutes, followed by a linear ramp of 10 K/min up to 200 °C, then held for 10 minutes. A deactivated transfer line with an inner diameter of 0.25 mm connected the GC to the headspace autosampler. Oven temperature was 85 °C, needle temperature 100 °C and transfer line temperature 110 °C. Carrier pressure was 32 psi and vial pressure 28.5 psi. Vial equilibration was set to 25 min, pressurisation time to 3 min, injection time to 0.4 min and dwell time to 0.5 min.

1.2 Modified Bradford protein assay

Microbial growth in the enrichment cultures was followed by measuring the protein concentration. Liquid samples (1-2 ml) were centrifuged at 6,000 × *g* for 10 min at 4 °C, then 500 µl lysis buffer (0.15 M NaOH and 0.45 % NaCl) was added to the pellet. The samples were incubated at 95 °C for 10 min and then centrifuged at 10,000 × *g* for 10 min at 4 °C. Depending on the protein concentration, 100 - 500 µl of the supernatant was mixed with Bradford reagent (PanReac AppliChem ITIW Reagents) to a final volume of 1 ml. Only the lysis buffer was used as blank. The mixture was vortexed and incubated for 2 min at room temperature in the dark. The samples were then transferred to cuvettes suitable for UV measurements (semi-micro 1.6 mL, 1 cm path length) and measured at 595 nm using a photometer (Spectrophotometer Genesys 10S UV-VIS; Thermo Fisher Scientific Inc.). The protein concentration was calculated using a calibration generated with bovine serum albumin as standard.

1.3 Sampling for gas composition analysis, and data analysis

Gas samples of 3 ml were taken from the headspace of a serum bottle with a nitrogen flushed syringe. The syringe was sealed with a rubber stopper immediately after sampling. The gas volumes of H_2, CO_2, CH_4 and N_2 were normalised to 100%, with the oxygen concentration set to be zero. It was assumed that observed traces of oxygen were caused by the transport of the sample to the device. This assumption was based on the observation that the indicator resazurin in the cultivation bottles ensured that no oxygen was present in the bottles.

1.4 Modified phenol-sulphuric acid method

The enrichment cultures were cultivated for either two (xylan enrichment cultures) or five weeks (Avicel® enrichment cultures) after completion of the enrichment process. The cultivation bottles were then opened and samples were taken while stirring the culture to ensure homogeneous samples.

Samples were centrifuged at 5,000 × *g* for 15 min at 4 °C. For measuring the cellulose content, the supernatant was discarded, the pellet was resuspended in deionised water, heated at 95 °C for 30 min and washed afterwards twice with deionised water (5,000 × *g* and 4 °C for 15 min). For measuring the soluble xylan, the supernatant of the sample was used. It was heated as described above, but the washing steps were not required. The pellet or supernatant was mixed with 1 ml of 65% sulphuric acid and incubated for 1 h. Samples were then diluted if necessary with deionised water and 200 µl of the liquid was mixed with 200 µl of 2% phenol and 1 ml of 98% sulphuric acid and incubated for 30 min at room temperature. The absorbance was then measured at 490 nm for determination of the Avicel® content or at 480 nm for xylan. Calibration curves were prepared with xylan (10-150 mg l^{-1}) or Avicel® (5-1,000 mg l^{-1}).

2. Production performance of enrichment cultures

2.1 Controls

Cultures without cellulose or hemicellulose but inoculated were treated the same way like the other cultures. These controls were carried along to test whether microorganisms were enriched on alternative carbon sources (not cellulose or xylan). In these control cultures, product formation was observed (Figures S1 and S2). In the first transfers, this could be explained by the presence of carbon sources contained in the inoculum. However, also in the later transfers, liquid and gas products were detected. Comparing these product concentrations with those of the cellulose enrichment cultures, the acetic acid production was in a similar range and also carbon dioxide was produced, though in smaller amounts (0.8 - 1.8% in the headspace). The only carbon sources in the medium that could have been used by the bacteria were cysteine or ascorbic acid. Therefore, SynCon2 medium lacking ascorbic acid, but still containing cysteine as reducing agent, was used for restarting enrichments based on cow manure and marshland soil. The control cultures with SynCon2 medium (without addition of cellulose or xylan) from marshland soil showed a decrease of acetic acid production, and the cultures based on cow manure showed almost no production after the restart (Figure S1).

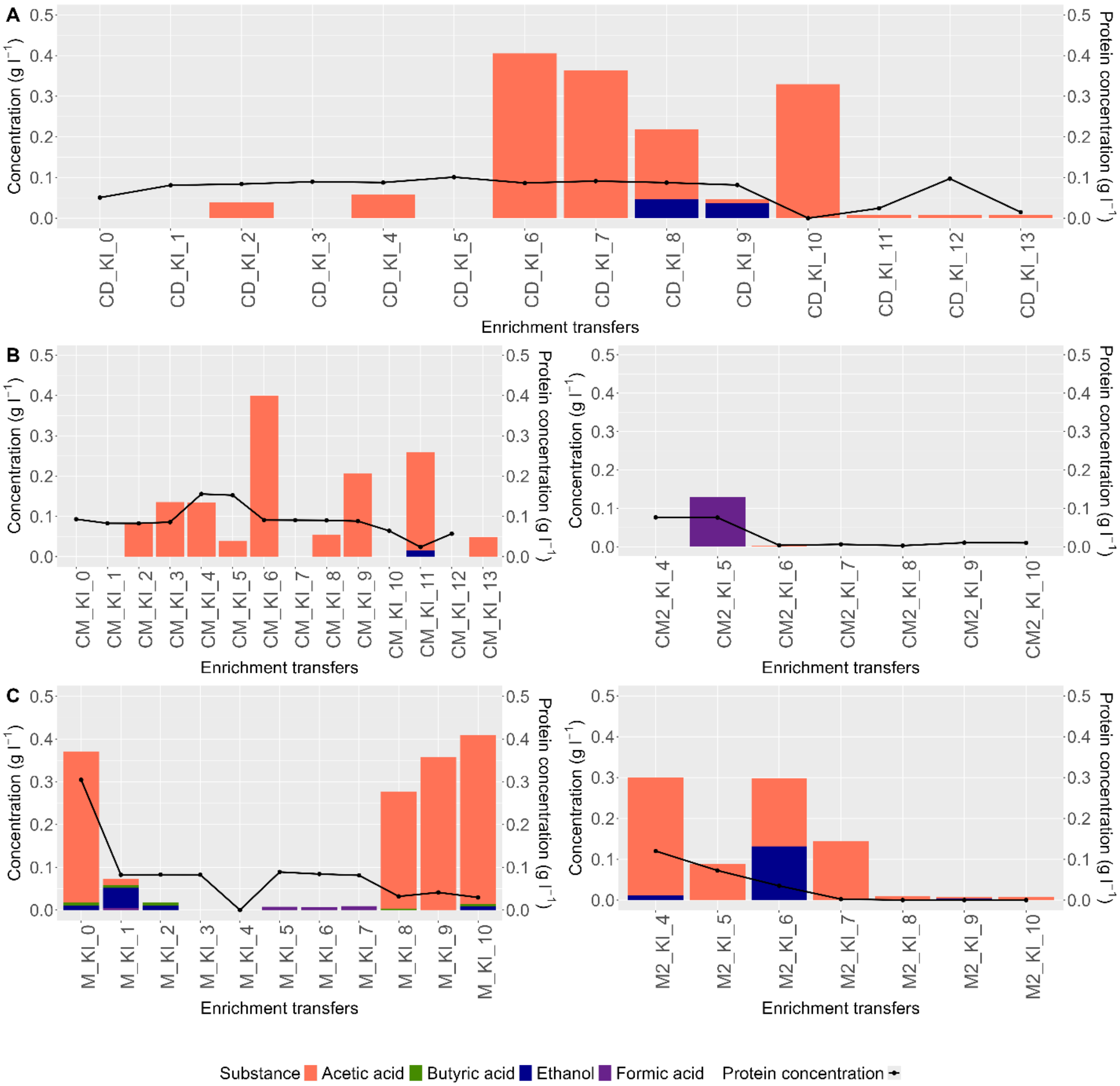

Figure 1. Liquid fermentation products and protein concentrations of the controls (KI) without addition of cellulose/hemicellulose are shown for the cultures from compost and digestate (A), from cow manure (B), and from marshland soil (C). On the right side (B-C), the production performance of the enrichment cultures restarted with SynCon2 is shown. CD, compost and digestate; CM, cow manure; CM2, cow manure restarted enrichment; M, marshland soil; M2, marshland soil restarted enrichment.

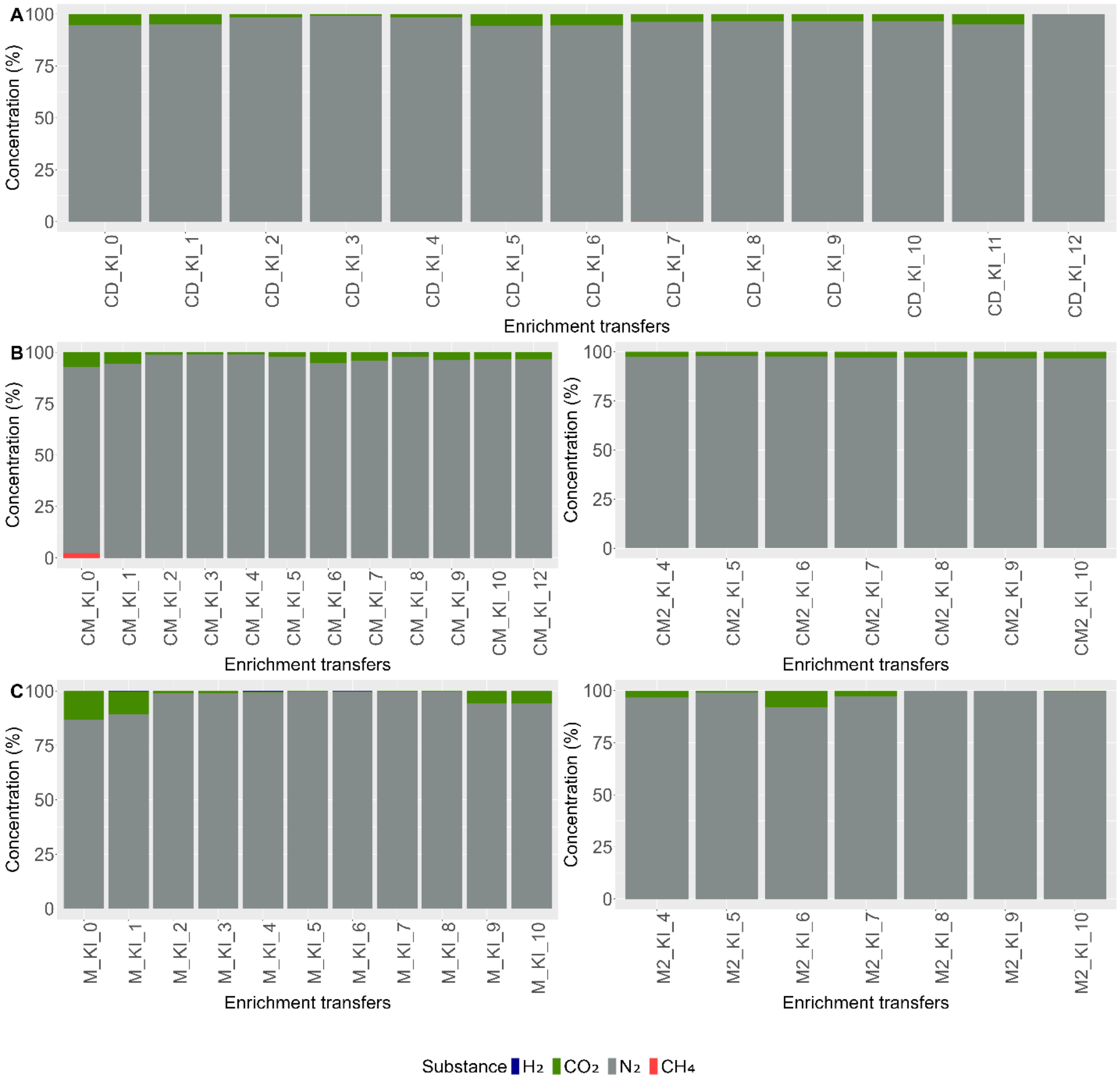

Figure 2. Gas composition of the controls (KI) without addition of cellulose/hemicellulose in the cultures from compost and digestate (A), from cow manure (B) and from marshland soil (C). On the right side (B-C), data from the enrichments restarted with SynCon2 is shown. CD, compost and digestate; CM, cow manure; CM2, cow manure restarted enrichment; M, marshland soil; M2, marshland soil restarted enrichment.

2.2 Relative gas composition of enrichment cultures from compost and digestate

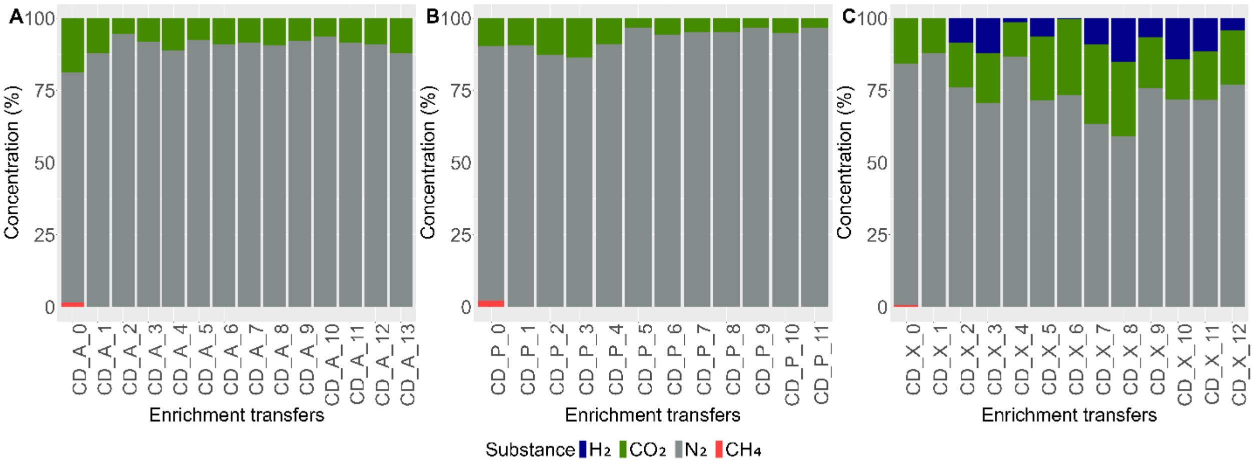

Figure 3. Gas composition of the enrichment cultures from compost and digestate with Avicel® (A), PASC (B), and xylan (C) in SynCon1 medium. Every bar represents a distinct enrichment transfer bottle.

The microorganisms of the cellulose enrichment cultures from compost and digestate produced only carbon dioxide with a proportion between 3.2% and 11% in the headspace. In the cultures with xylan, hydrogen was detected with proportions of up to 15% in the headspace in addition to carbon dioxide of up 28%.

2.3 Relative gas composition of enrichment cultures from cow manure

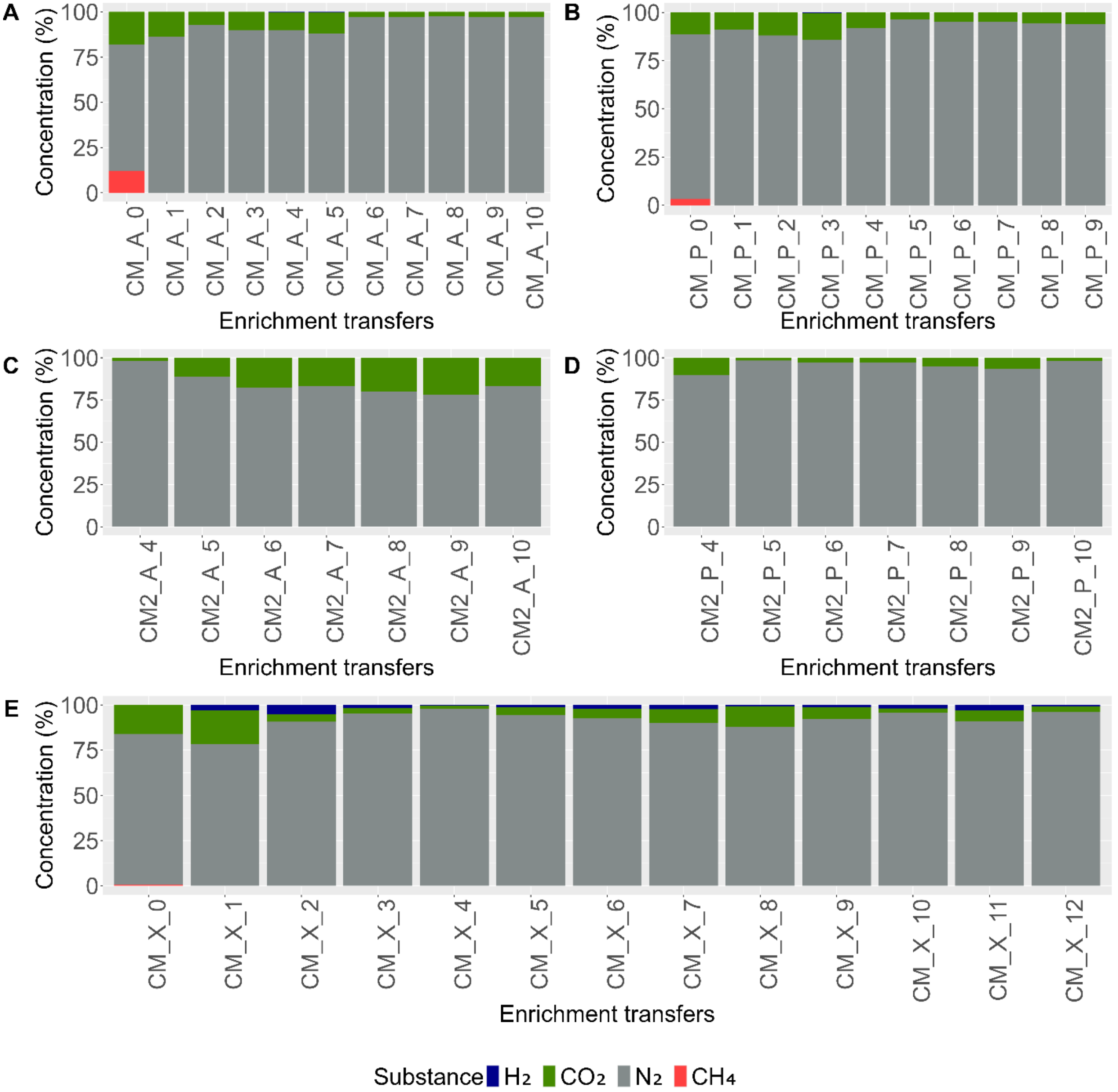

Figure 4. Gas composition of the enrichment cultures from cow manure with Avicel® (A and C), with PASC (B and D) and with xylan (E). SynCon1 medium was used in the cultures shown in A, B and E. Results of the cultures restarted with SynCon2 medium are shown in C and D. Every bar represents a distinct enrichment transfer bottle. CM, cow manure; CM2, cow manure restarted enrichment.

Carbon dioxide was the main gas component produced by the cultures from cow manure, and the gas composition barely changed. The addition of 2-bromoethanesulphonate in the first two transfers in the cellulose enrichment cultures was sufficient to inhibit further methane production. Hydrogen production was observed only in the xylan cultures, but in lower concentrations (1-5%) than in the cultures based on compost and digestate (15%; Figure S3).

2.4 Relative gas composition of the enrichment cultures from marshland soil

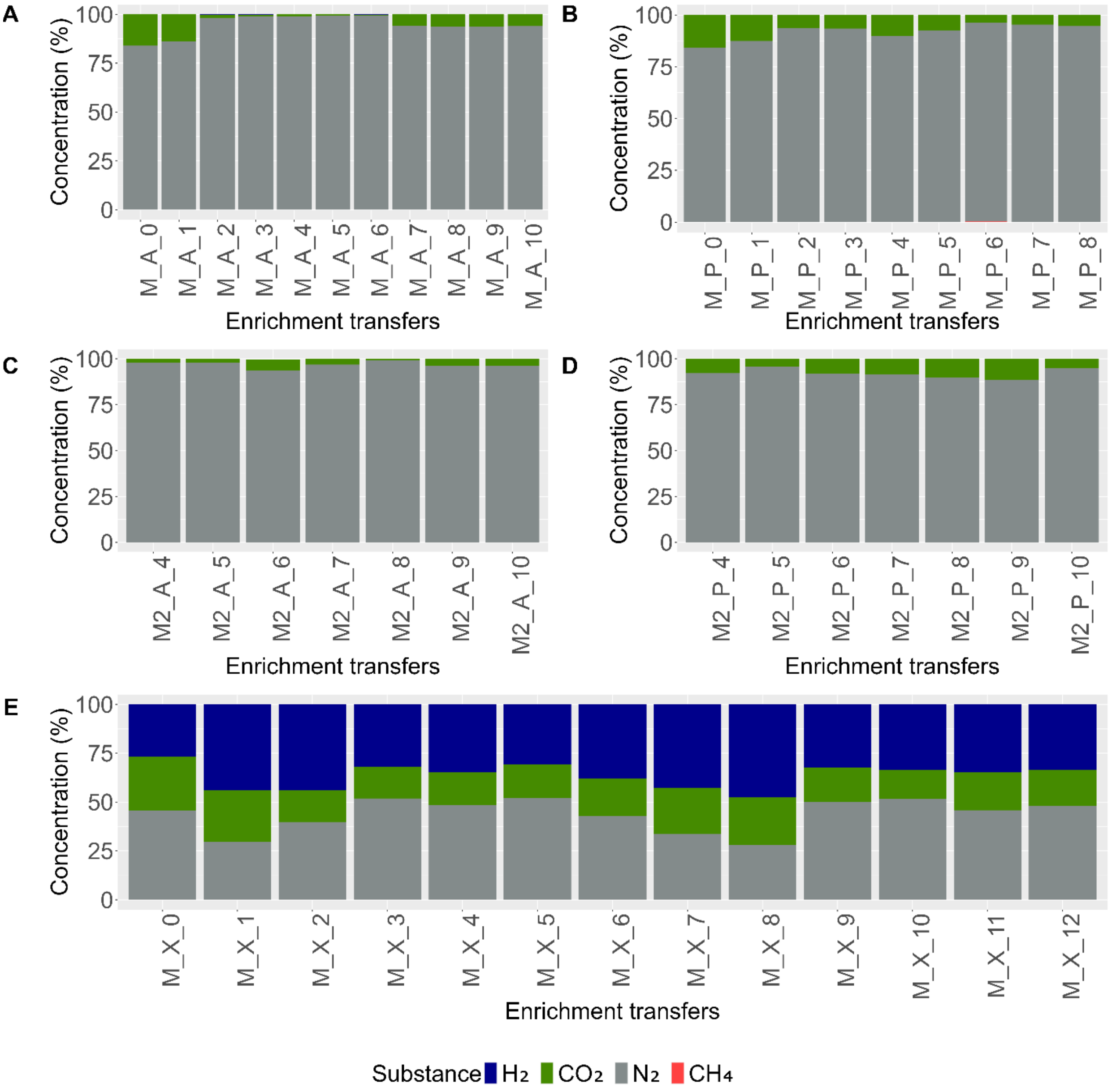

Figure 5. Gas composition of the enrichment cultures from marshland soil with Avicel® (A and C), with PASC (B and D) as well as with xylan (E). SynCon1 medium was used in the cultures shown in A, B and E. Results of the cultures restarted with SynCon2 medium are shown in C and D. Every bar represents a distinct enrichment transfer bottle. M, marshland soil; M2, marshland soil restarted enrichment.

The cultures with PASC or Avicel® from marshland soil produced only carbon dioxide during the enrichment. In contrast, high hydrogen concentrations of up to 47% were measured in the gas phase of the cultures with xylan. The addition of 2-bromoethanesulphonate was not necessary in any of the cultures, as no methane formation occurred.

2.5 Fermentation products and protein concentrations of cellulose enrichment cultures

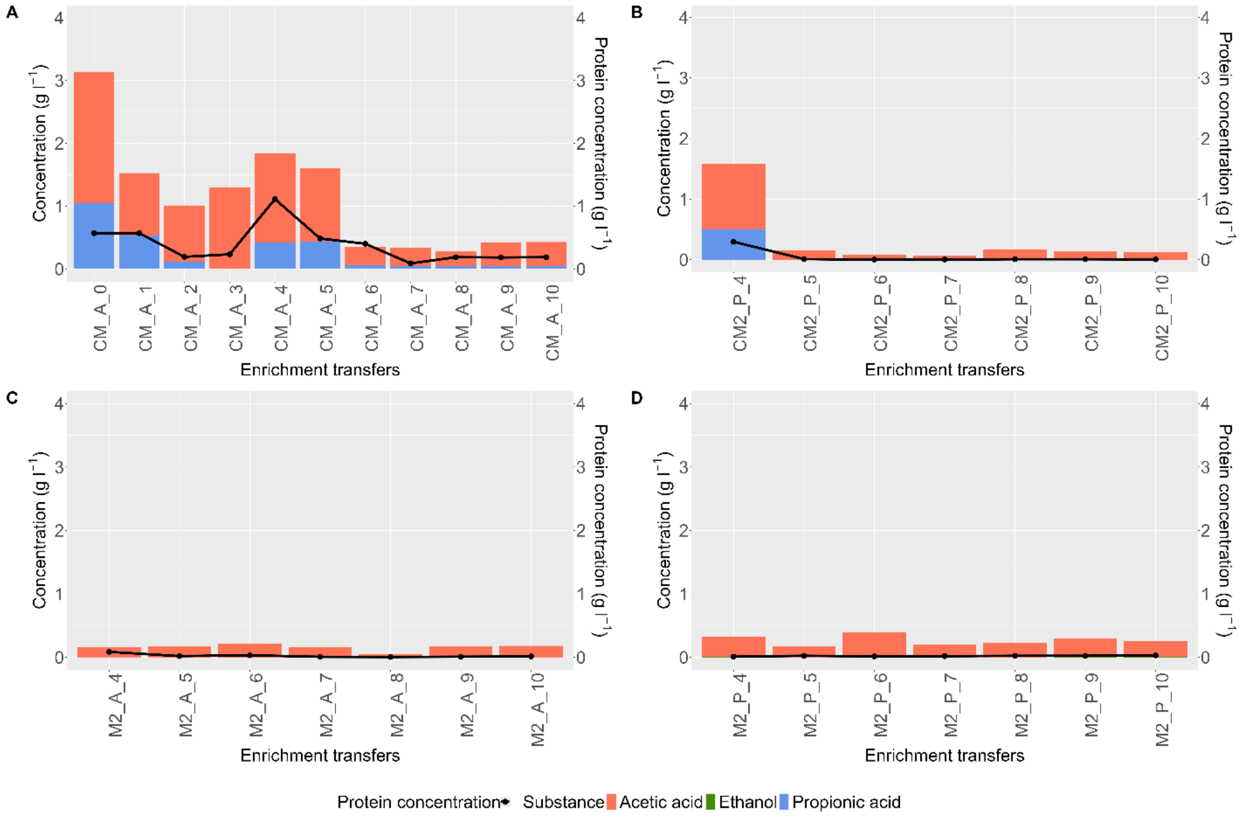

Figure 6. Liquid fermentation products and protein concentrations of the enrichment cultures from cow manure (A and B) and from marshland soil (C and D) with Avicel® (A and C) and with PASC (B and D) as substrate. Every bar represents a distinct enrichment transfer bottle. CM, cow manure; CM2, cow manure enrichment restarted with SynCon2; M2, marshland soil enrichment restarted with SynCon2.

2.6 Degradation of xylan or Avicel® in selected enrichment cultures

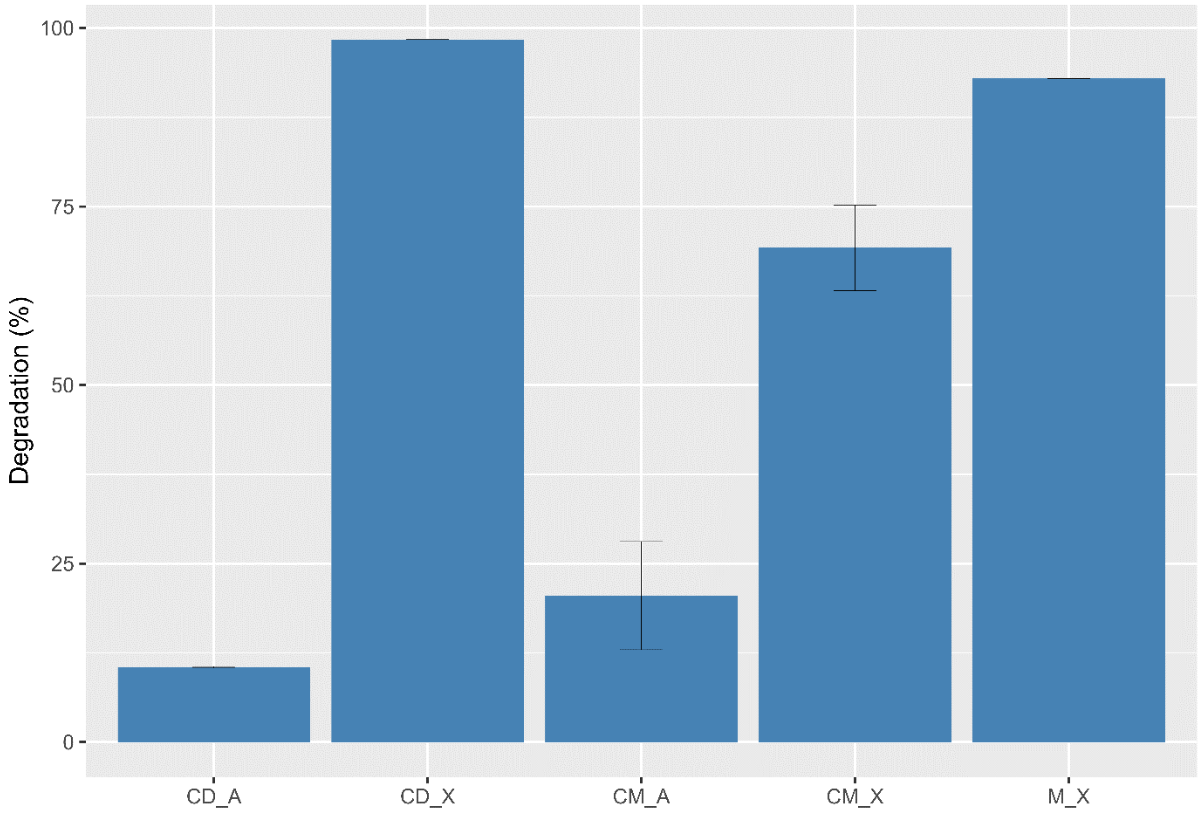

Figure 7. Degradation of xylan or Avicel® in selected enrichment cultures. Each bar represents the degradation of xylan after two weeks or the degradation of cellulose after five weeks of cultivation. Error bars correspond to the range of the measurement of two biological replicates for each bar. CD, compost and digestate; CM, cow manure; M, marshland soil; A, Avicel® as a substrate; X, xylan as a substrate.

3. Microbial community analysis

3.1 Spearman's correlation analysis

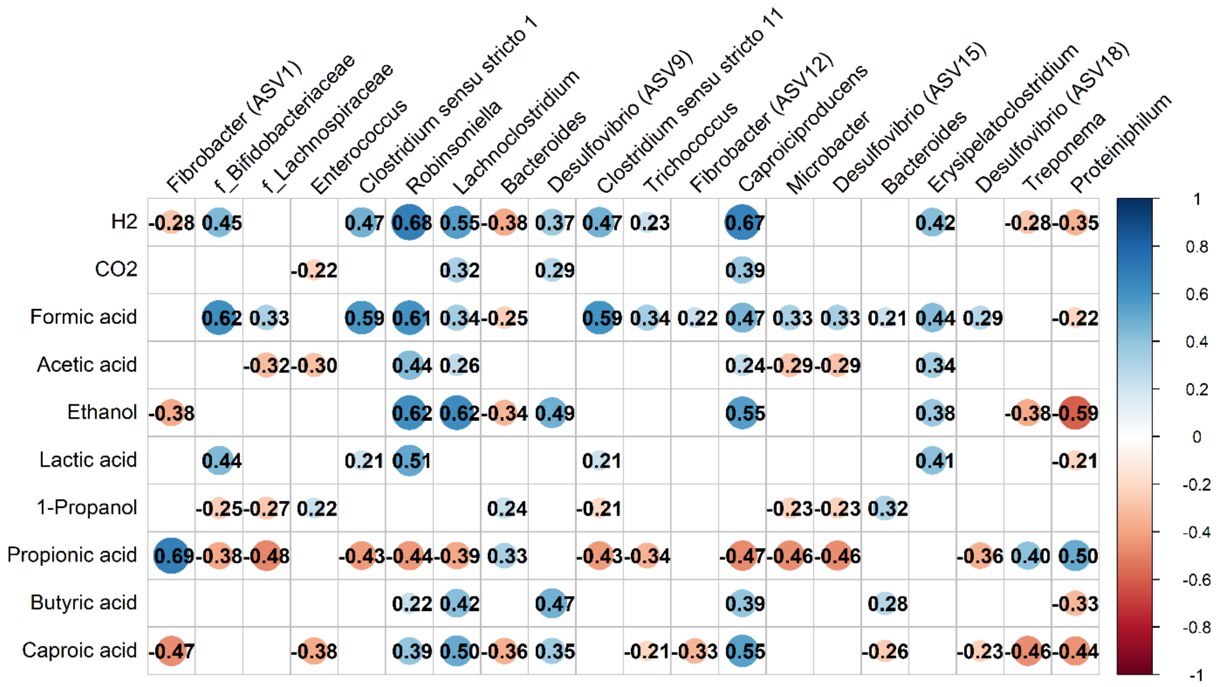

Figure 8. Spearman's correlation analysis with the top 20 amplicon sequence variants (ASVs) of the enrichment cultures. For the genera that are represented by more than one ASV, ASV number can be found in parentheses. Only statistically significant values ($p<0.05$) are shown.

3.2 Alpha- and beta-diversity analysis

Inoculum source and substrate influenced the community composition, with cultures enriched on Avicel® showing highest diversity, while cultures enriched from marshland soil having the lowest diversity.

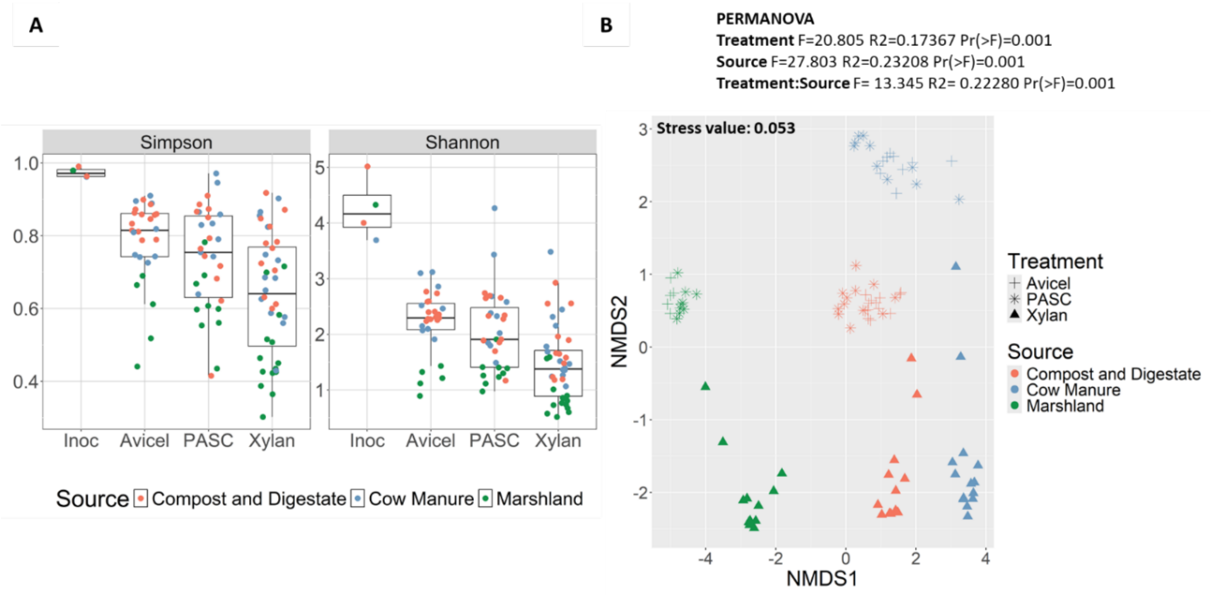

Figure 9. Alpha- and beta-diversity analysis of the inocula and enrichment cultures of the ASV. (A) Simpson and Shannon indices of the communities enriched from different inocula (Inoc) on Avicel®, PASC, or xylan as carbon source. (B) Non-metric multi-dimensional scaling (NMDS) ordination based on Bray-Curtis distance.

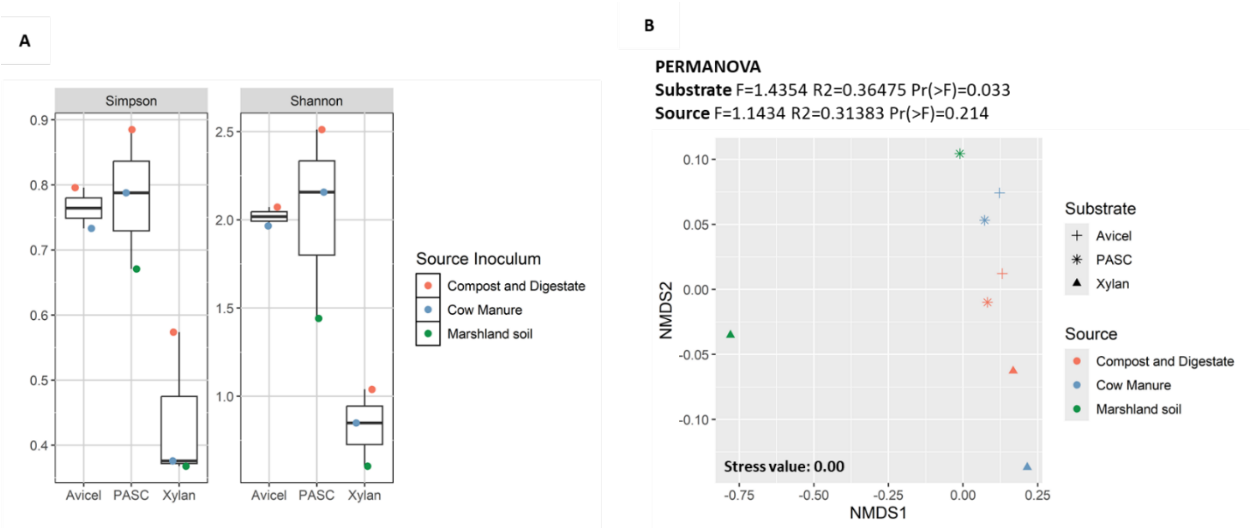

Figure 10. Alpha- and beta-diversity analysis of the enrichment cultures analysed by shotgun metagenome sequencing. (A) Simpson and Shannon indices of the communities enriched from different inocula on the substrates Avicel®, PASC, or xylan. (B) Non-metric multi-dimensional scaling (NMDS) ordination based on Bray-Curtis distance.

3.3 Community composition from amplicon sequencing data at phylum level

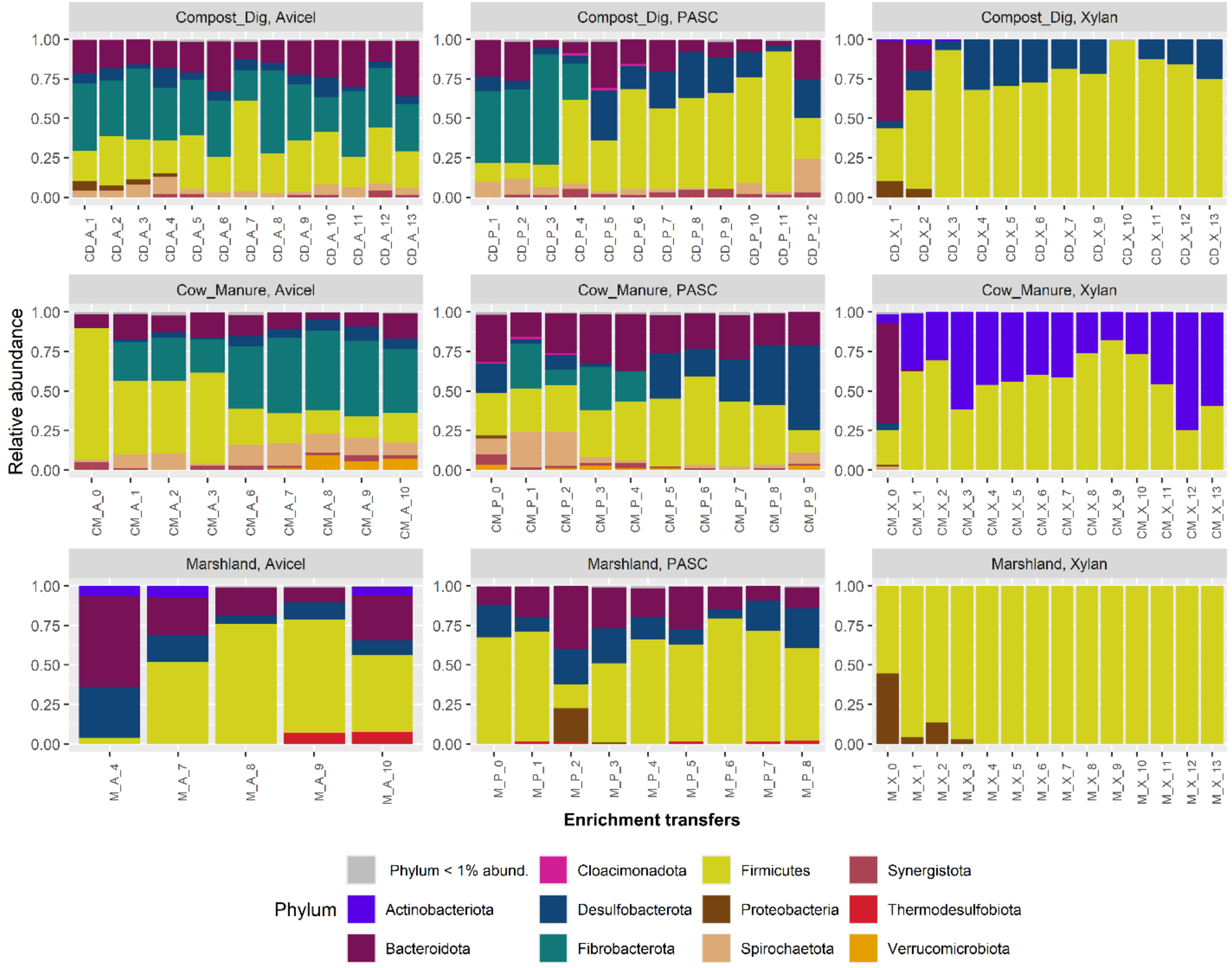

Figure 11. Community composition from amplicon sequencing data at phylum level of the enrichment cultures from different inoculum sources (compost and digestate, cow manure, and marshland soil) grown on Avicel®, PASC, or xylan. CD, compost and digestate; CM, cow manure; M, marshland soil; A, Avicel® as a substrate; X, xylan as a substrate.

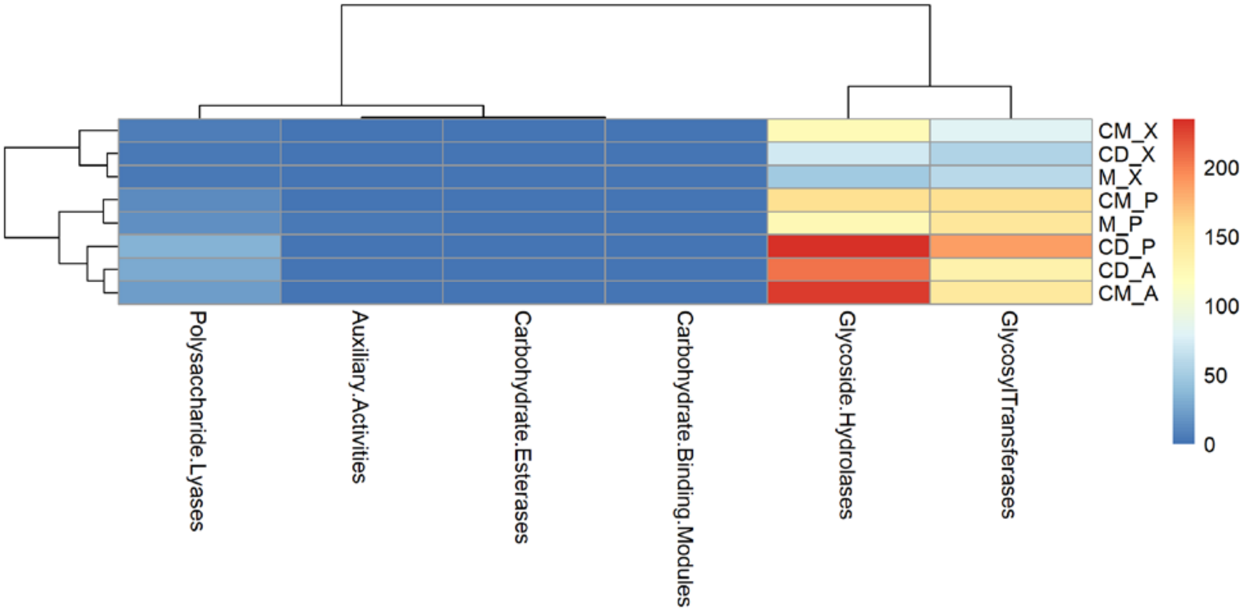

Figure 12. Heatmap of the genes found in the different enrichment cultures that could be classified in one of the six enzyme classes of CAZYme classification. CD, compost and digestate; CM, cow manure; M, marshland soil; A, Avicel® as a substrate; X, xylan as a substrate.

4. Metabolic modelling

4.1 Simulation results

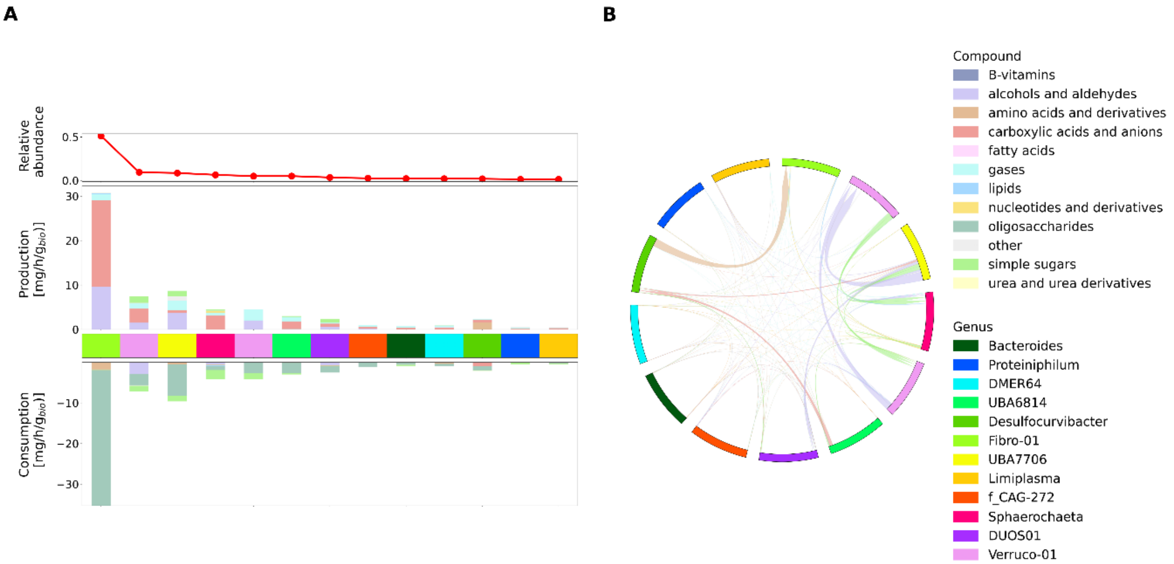

Figure 13. Simulation results for the cow manure culture enriched on Avicel®. Simulation was performed in SteadierCom with genome-scale metabolic models reconstructed based on the MAGs. Colours may denote multiple MAGs within the same genus. A) Relative abundance as well as consumption and production profiles of each community member, including the total amount of each compound class released and consumed from the growth medium and the amount shared with other members; B) cross-feeding interactions between community members (arrow thickness proportional to mass rate). All results represent the average flux distributions from 100 randomly sampled solutions (see Methods section 2.4 in the main manuscript) and are presented in rates of total mass exchanged (mg of compound per gram of community dry weight per hour). Only exchanges occurring in 10% or more of samples are shown. DMER64 belongs to the order Bacteroidales, UBA6814 belongs to the family Desulfovibrionaceae, UBA7706 belongs to the class Bacilli, f_CAG-727 belongs to the order Oscillospirales, and DUOS01 belongs to the family Treponemataceae.

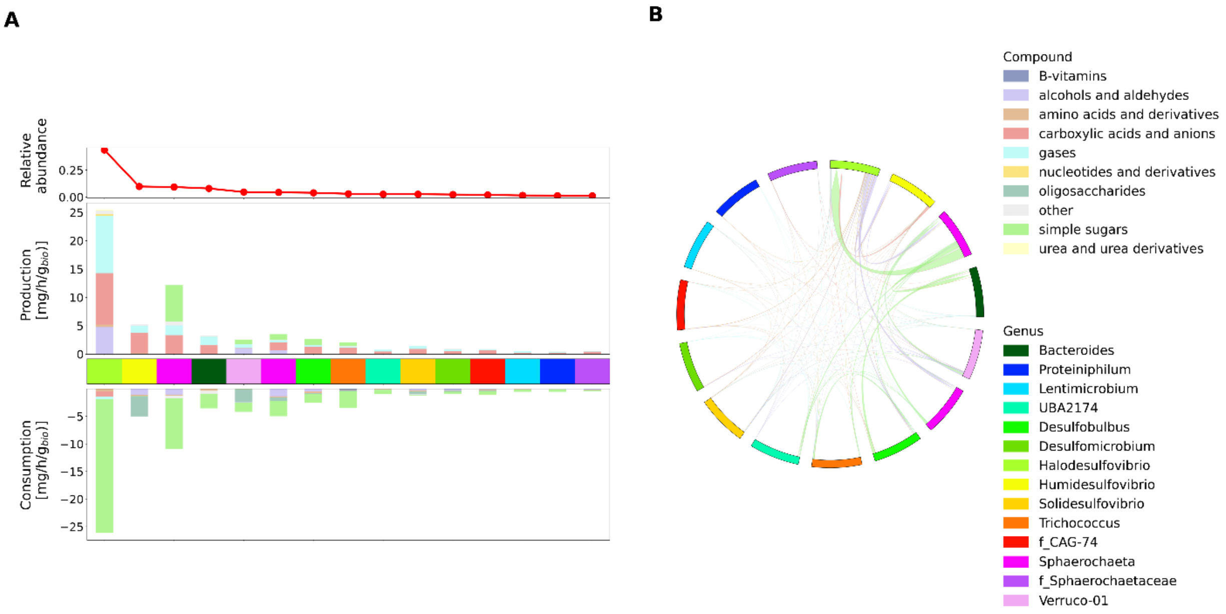

Figure 14. Simulation results for the cow manure culture enriched on PASC. Simulation was performed in SteadierCom with genome-scale metabolic models reconstructed based on the MAGs. Colours may denote multiple MAGs within the same genus. A) Relative abundance as well as consumption and production profiles of each community member, including the total amount of each compound class released and consumed from the growth medium and the amount shared with other members; B) cross-feeding interactions between community members (arrow thickness proportional to mass rate). All results represent the average flux distributions from 100 randomly sampled solutions (see Methods section 2.4 in the main manuscript) and are presented in rates of total mass exchanged (mg of compound per gram of community dry weight per hour). Only exchanges occurring in 10% or more of samples are shown. UBA2174 belongs to the order Desulfobacterales, and f_CAG-74 belongs to the order Christensenellales.

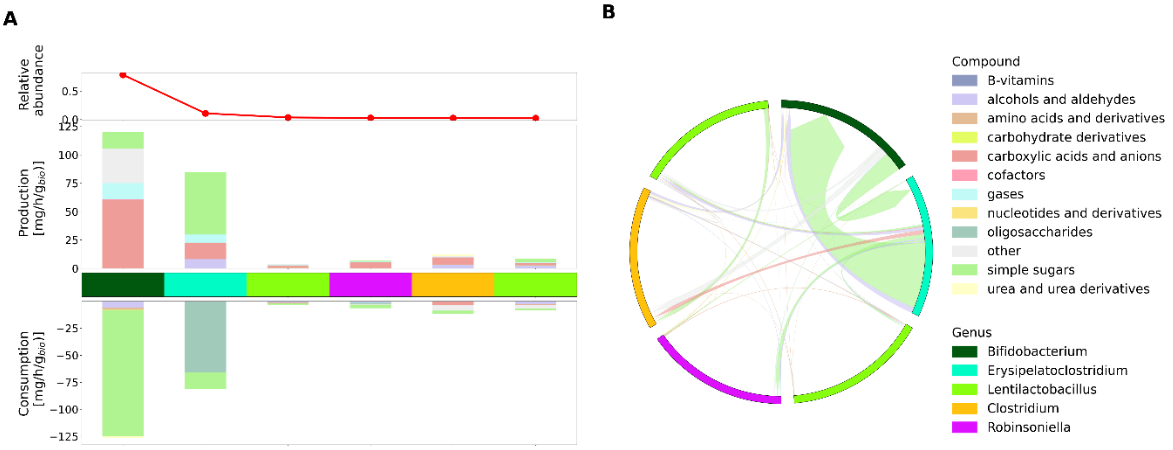

Figure 15. Simulation results for the cow manure culture enriched on xylan. Simulation was performed in SteadierCom with genome-scale metabolic models reconstructed based on the MAGs. Colours may denote multiple MAGs within the same genus. A) Relative abundance as well as consumption and production profiles of each community member, including the total amount of each compound class released and consumed from the growth medium and the amount shared with other members; B) cross-feeding interactions between community members (arrow thickness proportional to mass rate). All results represent the average flux distributions from 100 randomly sampled solutions (see Methods section 2.4 in the main manuscript) and are presented in rates of total mass exchanged (mg of compound per gram of community dry weight per hour). Only exchanges occurring in 10% or more of samples are shown.

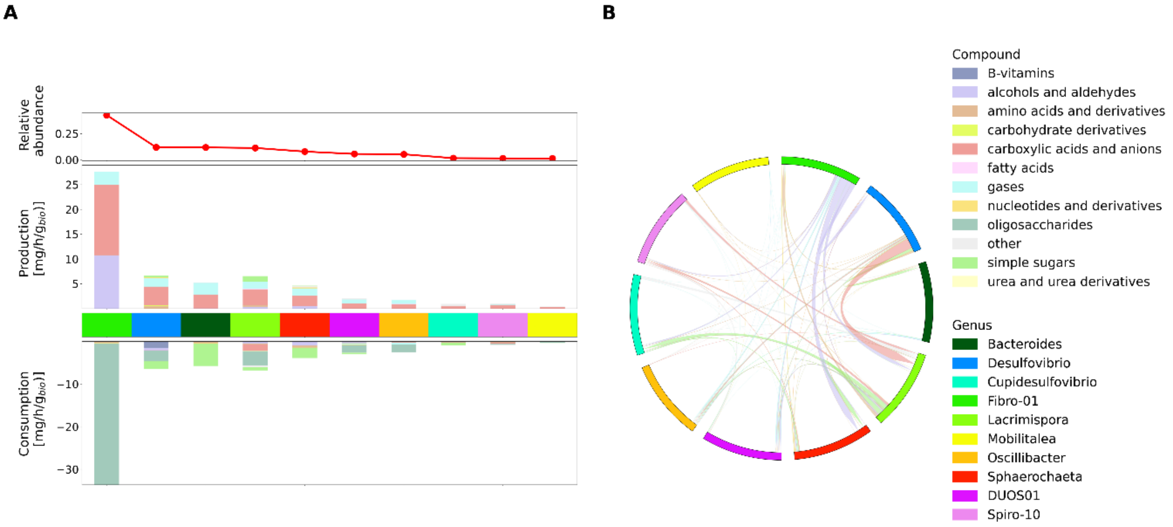

Figure 16. Simulation results for the compost and digestate culture enriched on Avicel®. Simulation was performed in SteadierCom with genome-scale metabolic models reconstructed based on the MAGs. Colours may denote multiple MAGs within the same genus. A) Relative abundance as well as consumption and production profiles of each community member, including the total amount of each compound class released and consumed from the growth medium and the amount shared with other members; B) cross-feeding interactions between community members (arrow thickness proportional to mass rate). All results represent the average flux distributions from 100 randomly sampled solutions (see Methods section 2.4 in the main manuscript) and are presented in rates of total mass exchanged (mg of compound per gram of community dry weight per hour). Only exchanges occurring in 10% or more of samples are shown. DUOS01 belongs to the family Treponemataceae.

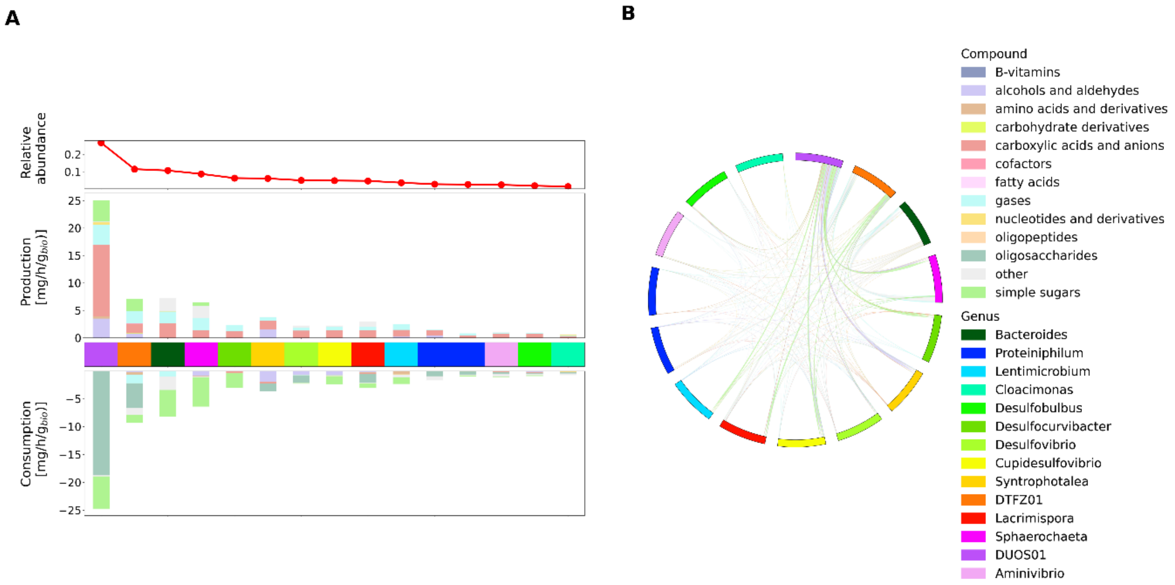

Figure 17. Simulation results for the compost and digestate culture enriched on PASC. Simulation was performed in SteadierCom with genome-scale metabolic models reconstructed based on the MAGs. Colours may denote multiple MAGs within the same genus. A) Relative abundance as well as consumption and production profiles of each community member, including the total amount of each compound class released and consumed from the growth medium and the amount shared with other members; B) cross-feeding interactions between community members (arrow thickness proportional to mass rate). All results represent the average flux distributions from 100 randomly sampled solutions (see Methods section 2.4 in the main manuscript) and are presented in rates of total mass exchanged (mg of compound per gram of community dry weight per hour). Only exchanges occurring in 10% or more of samples are shown. DTFZ01 belongs to the order Christensenellales. DUOS01 belongs to the family Treponemataceae.

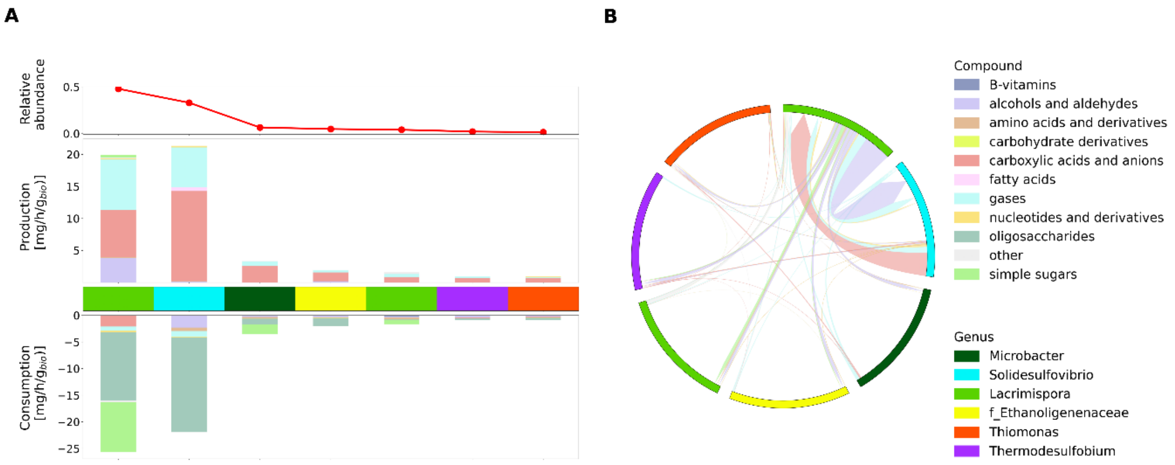

Figure 18. Simulation results for the marshland soil culture enriched on PASC. Simulation was performed in SteadierCom with genome-scale metabolic models reconstructed based on the MAGs. Colours may denote multiple MAGs within the same genus. A) Relative abundance as well as consumption and production profiles of each community member, including the total amount of each compound class released and consumed from the growth medium and the amount shared with other members; B) cross-feeding interactions between community members (arrow thickness proportional to mass rate). All results represent the average flux distributions from 100 randomly sampled solutions (see Methods section 2.4 in the main manuscript) and are presented in rates of total mass exchanged (mg of compound per gram of community dry weight per hour). Only exchanges occurring in 10% or more of samples are shown.

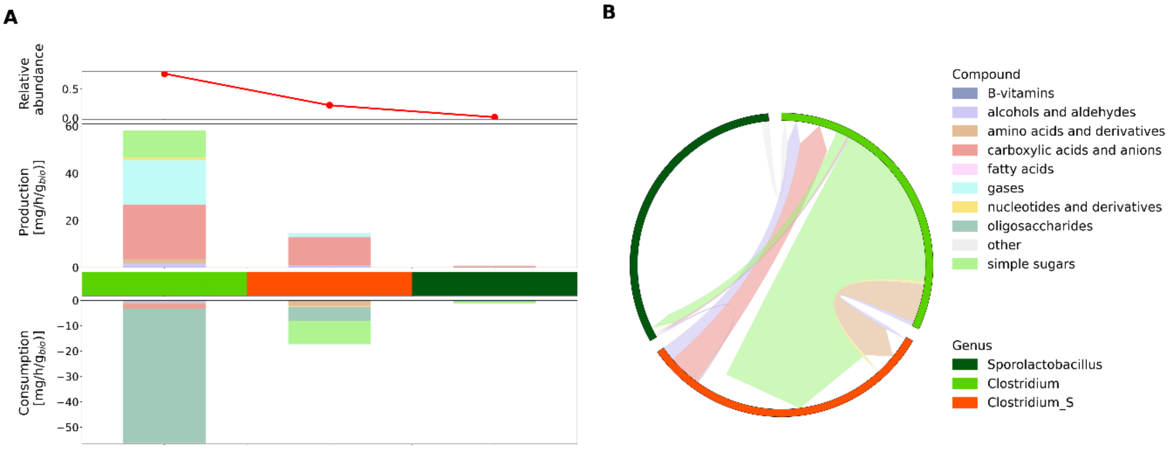

Figure 19. Simulation results for the marshland soil culture enriched on xylan. Simulation was performed in SteadierCom with genome-scale metabolic models reconstructed based on the MAGs. Colours may denote multiple MAGs within the same genus. A) Relative abundance as well as consumption and production profiles of each community member, including the total amount of each compound class released and consumed from the growth medium and the amount shared with other members; B) cross-feeding interactions between community members (arrow thickness proportional to mass rate). All results represent the average flux distributions from 100 randomly sampled solutions (see Methods section 2.4 in the main manuscript) and are presented in rates of total mass exchanged (mg of compound per gram of community dry weight per hour). Only exchanges occurring in 10% or more of samples are shown.

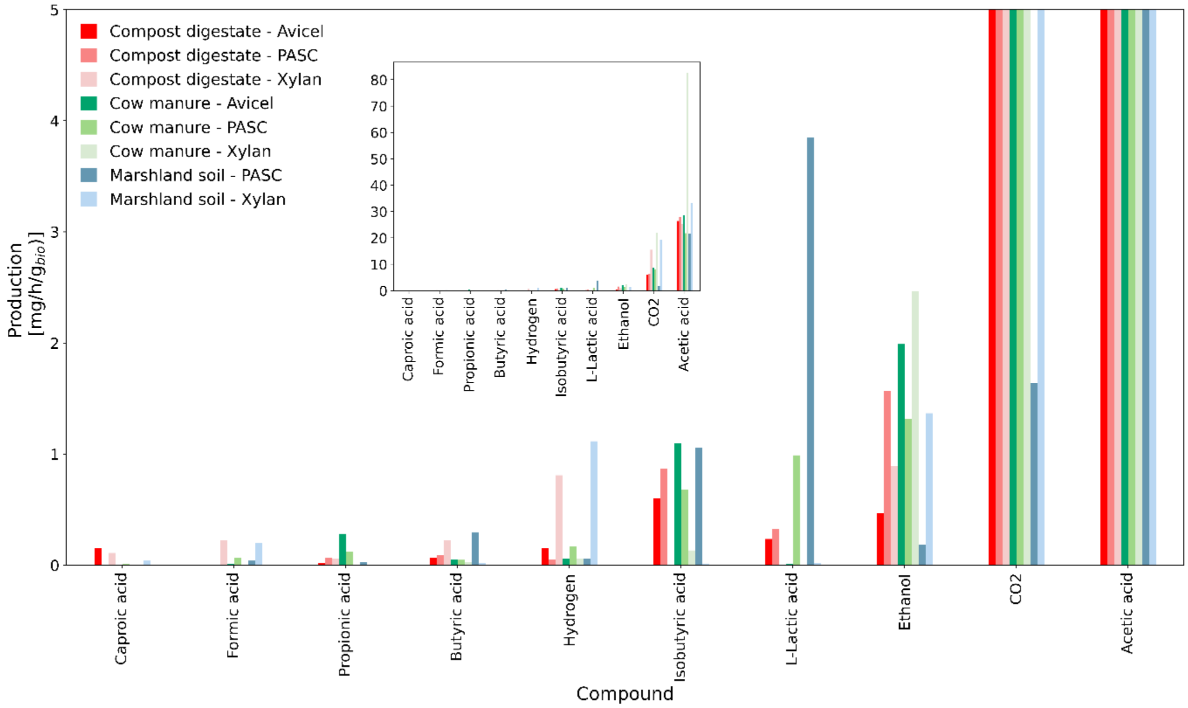

Figure 20. Simulation results for the production of all experimentally measured compounds. For each community, the values represent the average across 100 randomly sampled solutions.

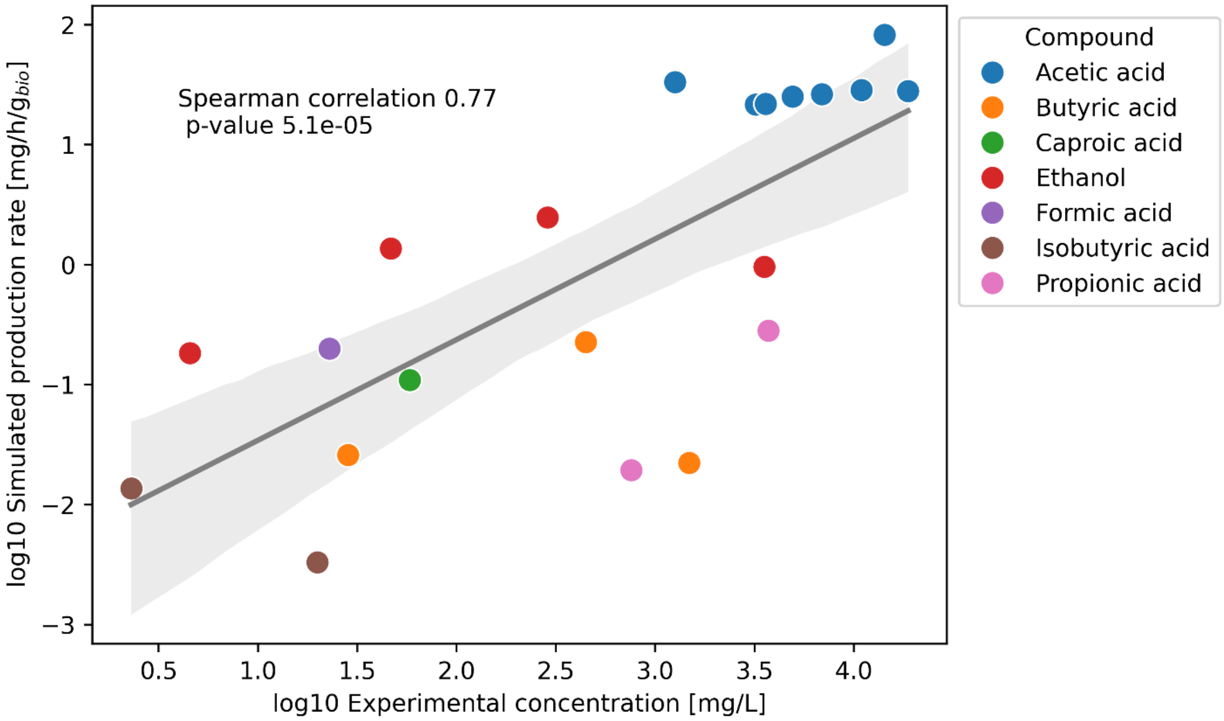

Figure 21. Spearman correlation analysis of simulated production rate and experimentally measured metabolite concentration.

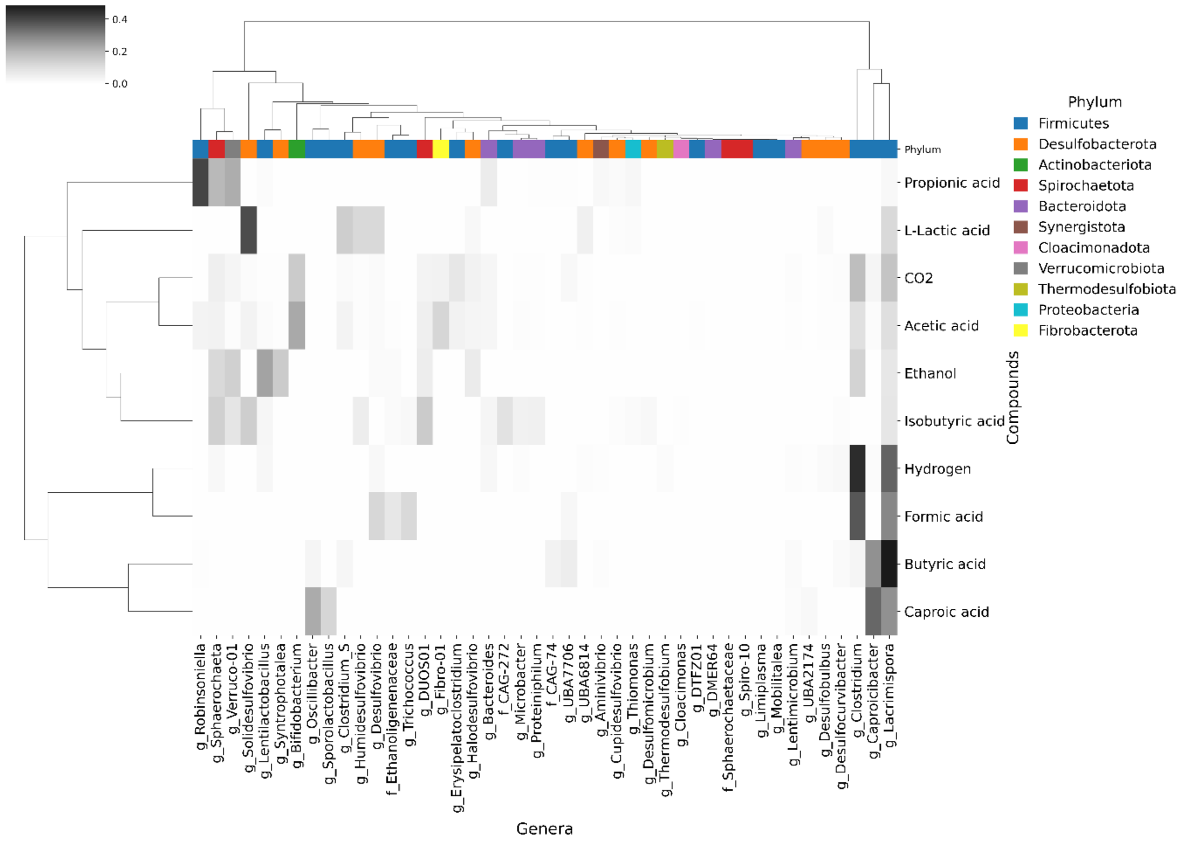

Figure 22. Clustering of predicted production profiles per genus. The total production rates across communities are added and grouped by genera. The final values are normalised per compound (i.e., total production of a given genus divided by total production across genera for a given compound). Sulphate reducers are indicated as lactate producers, which reflects limitations due to misannotations and gap-filling in the models (see section 4.2).

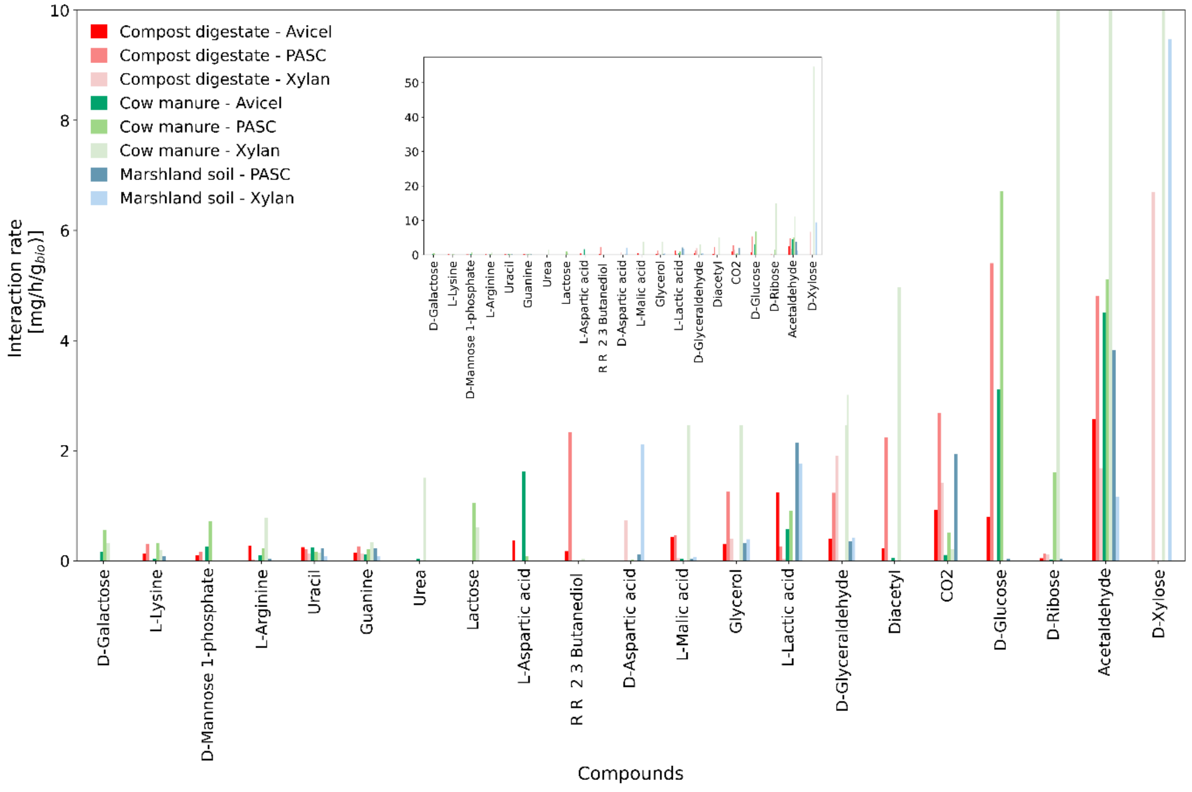

Figure 23. Most compounds participating in cross-feeding interactions based on total exchange rate. Only compounds with a total exchange rate above 1 mg/g dry weight/h and a frequency above 10% in the random sampling are shown.

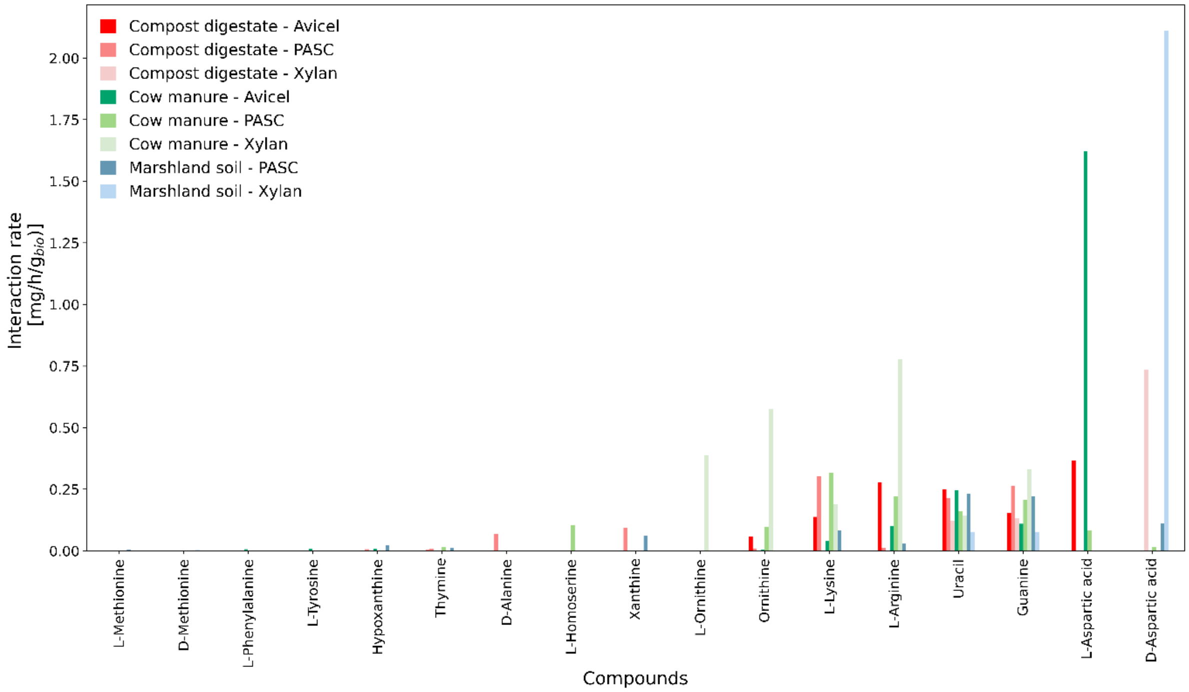

Figure 24. Most compounds participating in cross-feeding interactions based on total exchange rate (limited to the following compound classes: nucleotides, amino acids, B-vitamins, and cofactors). Only compounds with a total exchange rate above 1 mg/g dry weight/h and a frequency above 10% in the random sampling are shown.

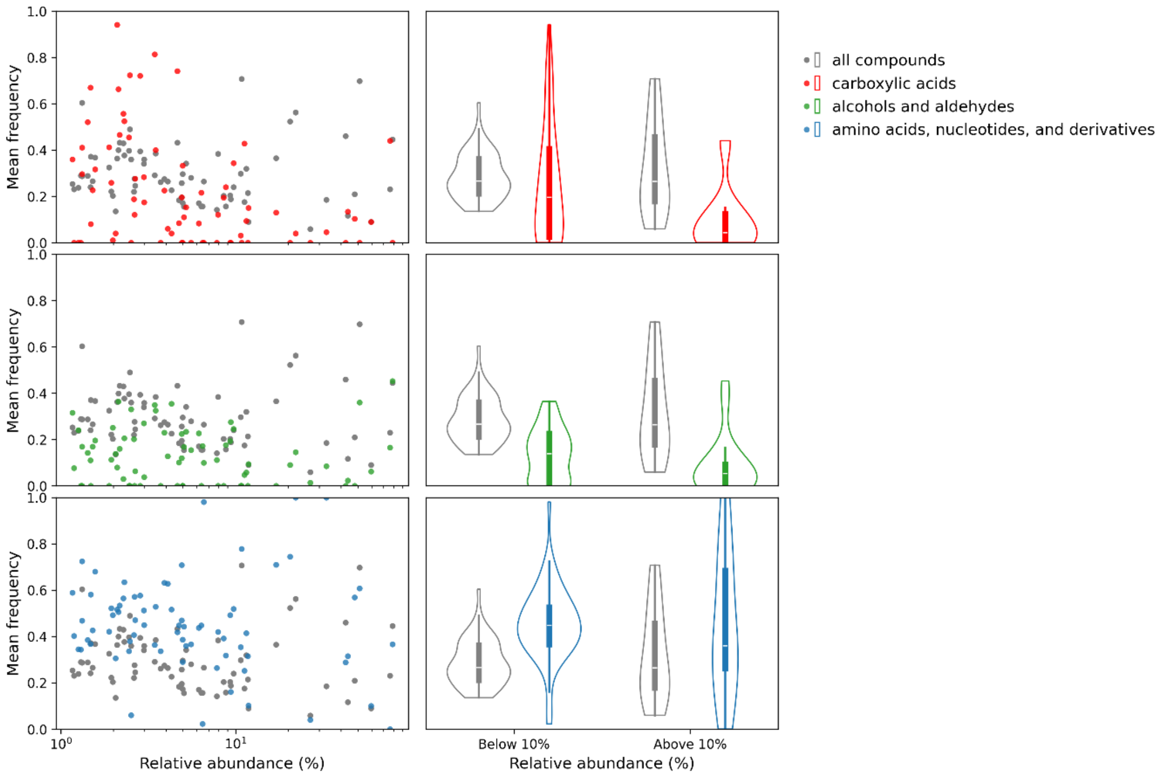

Figure 25. Scatter and violin plot showing the simulated frequency of consumption of different classes of compounds for the bacterial members in the enrichment cultures (one point in the scatter plot corresponds to one member). The value is a representative of the bacterium's dependency on the compounds from the compound class, as those consuming a compound more frequently are like to be more dependent on the compound. The grey elements show the average for all compounds (all figures), red elements show the average for carboxylic acids (top figure), green elements show the average for alcohols and aldehydes (middle figure), and blue elements show the average for amino acids, nucleotides, and derivatives (bottom figure).

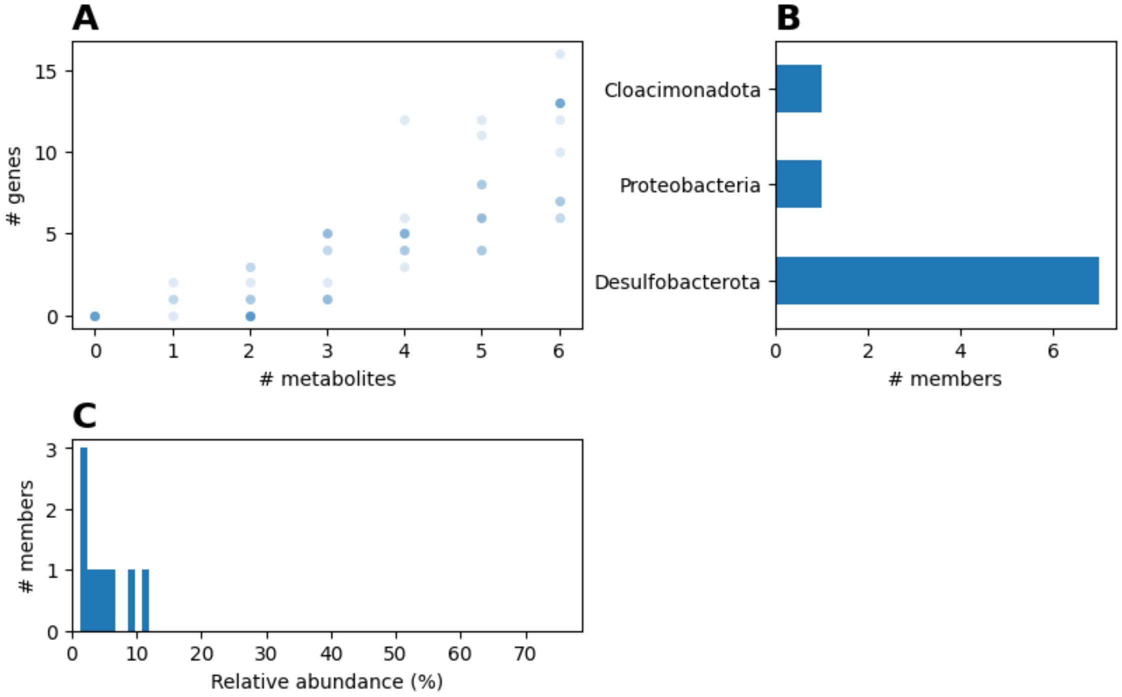

Figure 26. (A) Scatter plot showing the number of oligosaccharide metabolites in the models, from xylan and cellulose with three sugar units or more. The x-axis shows the number of metabolites and the y-axis shows the number of genes associated with reactions connected to the metabolites. (B) and (C) show bacterial members of the enrichment cultures that have metabolites and reactions for processing at least one oligosaccharide in their model, but lack associated genes. These members are grouped by phylum in the bar plot (B) and by relative abundance in the histogram (C).

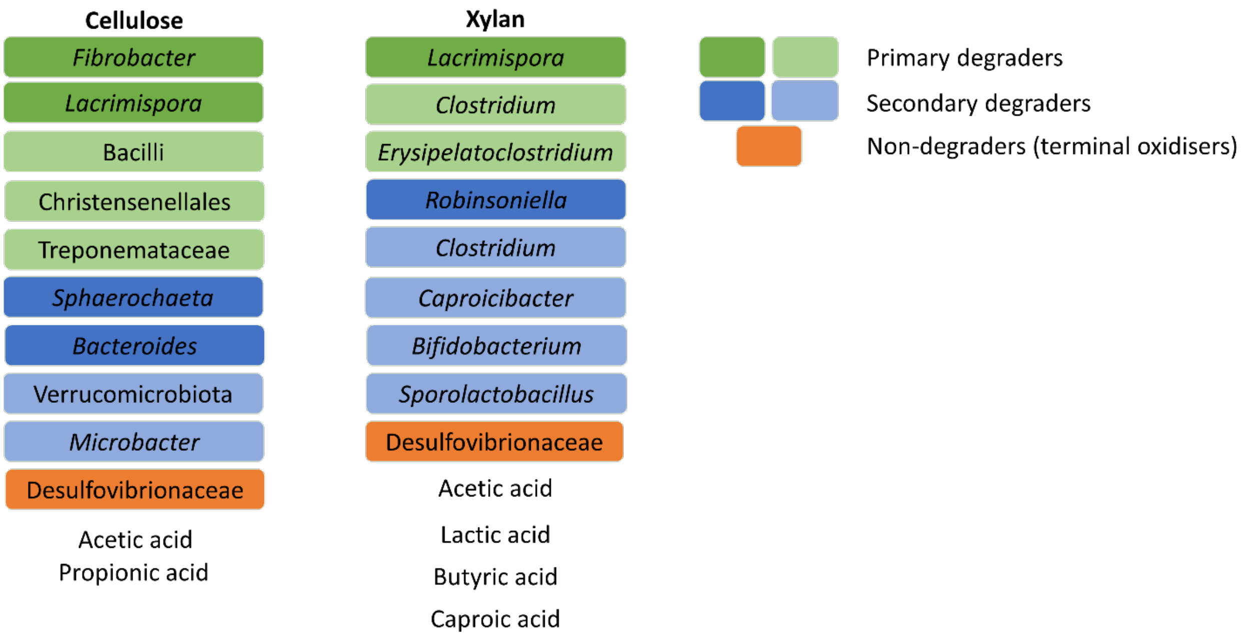

Figure 27. Schematic representation of the dominant members of trophic guilds and their associated products in cellulose and xylan cultures. Note that *Caproicibacter* is also referred to as *Caproiciproducens* in some databases, reflecting differences in taxonomic annotation.

4.2 Limitations and contradicting results

Compounds that are exchanged in smaller amounts, such as nucleotides, amino acids, and vitamins, are also known to play important roles in syntrophic relationships between bacteria [1]. A closer inspection of these compound classes (Figure S23) shows that aspartate, guanine, uracil, arginine, and lysine are the most exchanged (by total mass). We found that members of the Lachnospiraceae were the most frequent donors of guanine (odds ratio 5.8, adjusted p-value < 0.05) and recipients of thymine in cellulose-based cultures (odds ratio 9.0, adjusted p-value < 0.05). However, in general, we found only a small number of significant associations, most likely due to the limited sample size.

Solidesulfovibrio was identified as the main L-lactic acid producer across all communities (Figure S22), especially in marshland soil cultures with PASC (Figure S18), which contradicts literature data about sulphate-reducing bacteria[2]. Also, gene-reaction-metabolite analysis for oligosaccharide metabolism shows a positive trend between gene associations and metabolite-reaction presence (Figure S26A). However, particularly Desulfobacterota and low abundant genera were predicted to contain these metabolites and reactions without genetic association (Figure S26B, S26C) and to consume oligosaccharides in the simulation, due to gap-filling in oligosaccharide and sugar metabolism. The few gene annotations in this pathway for low abundance members, and their frequent consumption of carboxylic acids, alcohols and aldehydes could point to a higher dependency on fermentation products in comparison to more abundant members.

5. References

1. D’Souza, G. *et al.* Less is more: selective advantages can explain the prevalent loss of biosynthetic genes in bacteria. *Evolution; international journal of organic evolution* **68,** 2559–2570; 10.1111/evo.12468 (2014).
2. Barton, L. L. & Hamilton, W. A. *Sulphate-Reducing Bacteria* (Cambridge University Press, 2009).
